# Machine learning-enabled prediction of prolonged length of stay in hospital after surgery for tuberculosis spondylitis patients with unbalanced data: a novel approach using explainable artificial intelligence (XAI)

**DOI:** 10.1186/s40001-024-01988-0

**Published:** 2024-07-25

**Authors:** Parhat Yasin, Yasen Yimit, Xiaoyu Cai, Abasi Aimaiti, Weibin Sheng, Mardan Mamat, Mayidili Nijiati

**Affiliations:** 1 Department of Spine Surgery, The Sixth Affiliated Hospital of Xinjiang Medical University, Urumqi, 830000 Xinjiang People’s Republic of China; 2 Department of Spine Surgery, The First Affiliated Hospital of Xinjiang Medical University, Urumqi, 830054 Xinjiang People’s Republic of China; 3 Department of Radiology, The First People’s Hospital of Kashi Prefecture, Kashi, 844000 Xinjiang People’s Republic of China; 4 Department of Anesthesiology, The First Affiliated Hospital of Xinjiang Medical University, Urumqi, 830054 Xinjiang People’s Republic of China; 5 Department of Radiology, The Fourth Affiliated Hospital of Xinjiang Medical University(Xinjiang Hospital of Traditional Chinese Medicine), Urumqi, 830002 Xinjiang People’s Republic of China; 6 Xinjiang Key Laboratory of Artificial Intelligence Assisted Imaging Diagnosis, Kashi, 844000 Xinjiang People’s Republic of China

**Keywords:** Machine learning, Tuberculosis spondylitis, Explainable artificial intelligence, Imbalanced data

## Abstract

**Background:**

Tuberculosis spondylitis (TS), commonly known as Pott’s disease, is a severe type of skeletal tuberculosis that typically requires surgical treatment. However, this treatment option has led to an increase in healthcare costs due to prolonged hospital stays (PLOS). Therefore, identifying risk factors associated with extended PLOS is necessary. In this research, we intended to develop an interpretable machine learning model that could predict extended PLOS, which can provide valuable insights for treatments and a web-based application was implemented.

**Methods:**

We obtained patient data from the spine surgery department at our hospital. Extended postoperative length of stay (PLOS) refers to a hospitalization duration equal to or exceeding the 75th percentile following spine surgery. To identify relevant variables, we employed several approaches, such as the least absolute shrinkage and selection operator (LASSO), recursive feature elimination (RFE) based on support vector machine classification (SVC), correlation analysis, and permutation importance value. Several models using implemented and some of them are ensembled using soft voting techniques. Models were constructed using grid search with nested cross-validation. The performance of each algorithm was assessed through various metrics, including the AUC value (area under the curve of receiver operating characteristics) and the Brier Score. Model interpretation involved utilizing methods such as Shapley additive explanations (SHAP), the *Gini* Impurity Index, permutation importance, and local interpretable model-agnostic explanations (LIME). Furthermore, to facilitate the practical application of the model, a web-based interface was developed and deployed.

**Results:**

The study included a cohort of 580 patients and 11 features include (CRP, transfusions, infusion volume, blood loss, X-ray bone bridge, X-ray osteophyte, CT-vertebral destruction, CT-paravertebral abscess, MRI-paravertebral abscess, MRI-epidural abscess, postoperative drainage) were selected. Most of the classifiers showed better performance, where the XGBoost model has a higher AUC value (0.86) and lower Brier Score (0.126). The XGBoost model was chosen as the optimal model. The results obtained from the calibration and decision curve analysis (DCA) plots demonstrate that XGBoost has achieved promising performance. After conducting tenfold cross-validation, the XGBoost model demonstrated a mean AUC of 0.85 ± 0.09. SHAP and LIME were used to display the variables’ contributions to the predicted value. The stacked bar plots indicated that infusion volume was the primary contributor, as determined by Gini, permutation importance (PFI), and the LIME algorithm.

**Conclusions:**

Our methods not only effectively predicted extended PLOS but also identified risk factors that can be utilized for future treatments. The XGBoost model developed in this study is easily accessible through the deployed web application and can aid in clinical research.

**Supplementary Information:**

The online version contains supplementary material available at 10.1186/s40001-024-01988-0.

## Introduction

Spinal infection (SI) is a condition that occurs when the vertebral body, intervertebral disc, and surrounding paraspinal tissue become infected [1]. Tuberculous spondylitis (TS), commonly known as Pott’s disease, is a common cause of SI. While rarely seen in developed countries, it remains a significant public health concern in developing nations [2]. TS accounts for approximately 50% of extrapulmonary musculoskeletal tuberculosis cases and is one of the most frequent forms of skeletal tuberculosis [3]. Incidence rates of TS have increased in developing countries in recent years. Diagnosis of TS typically involves laboratory, radiology, and histopathology evaluations. Tuberculosis may lead to a kyphotic deformity of the spine associated with paralysis. The management strategy for tuberculous spondylitis (TS) is contingent upon the extent of the disease and the identification of neurological deficits through thorough clinical examination. Treatment for TS primarily involves adequate antituberculous drugs with or without surgical interventions to ensure sufficient decompression, debridement, and spinal stability [4]. Nonetheless, surgical treatment rates for TS persist at high levels, particularly in developing countries [5]. As a result, hospitalization-related healthcare expenses remain a significant healthcare burden for both patients and healthcare systems.

The escalation of healthcare costs in nearly all countries, particularly those that are developing, has emerged as a pressing social issue. One of the urgent challenges that require attention is the problem of extended hospital stays. The length of stay (LOS) after surgery, which denotes the duration of hospitalization, is widely considered a key indicator of efficiency and a proxy for the utilization of hospital resources [6]. Moreover, understanding LOS can facilitate improved comprehension of patient flow patterns, which are essential for comprehending the operational and clinical functions of healthcare systems [7]. More critically, diminishing LOS can potentially contribute to mitigating costs and enhancing patient outcomes. Consequently, there is a growing interest in developing robust predictive models of LOS. However, previous researches mainly focus on impacts of the degenerative diseases or the procedure approach selection on the LOS after surgery, resorting to conventional statistical analysis that was inferior to the performances of all other machine learning techniques when decision-making in clinical practical [8, 9].

The duration of hospitalization, commonly known as length of stay (LOS), is associated with a range of health information, including diagnoses, lab results, clinical data, and demographics. The integration of multiple biological information sources into a statistical learning framework represents a valuable application of machine learning (ML) algorithms. ML models can handle a diverse array of data types, including sociodemographic information, symptoms, laboratory tests, radiology tests, and others, which can significantly enhance classification accuracy [10]. Nonetheless, ML models often operate as black boxes, rendering the interpretation of their predictions difficult, particularly for complex models. To mitigate this limitation, several explainable artificial intelligence (XAI) techniques have been introduced to elucidate how these models function [11]. An effective approach involves employing the additive feature attribution method, wherein adjustments are made to input variables to evaluate the individual contribution of each feature concerning the model’s predictive outcomes. Given the critical nature of clinical decisions, understanding how models make predictions is paramount. Toward this end, the local interpretable model-agnostic explanation (LIME) approximates black-box models with locally generated surrogate models [12]. Moreover, the Shapley additive explanation (SHAP), a recent XAI technique, offers a more comprehensive perspective of each feature’s contribution to predicting clinical outcomes [13]. Applying SHAP to our analysis, we identified the features with the greatest predictive power to delineate between target and non-target groups.

The issue of extended hospitalization poses a significant challenge for patients residing in underdeveloped areas, necessitating the development of accurate predictive models that rely on readily available data sourced from routine primary care. To address this challenge, advanced data mining techniques, predominantly machine learning (ML), are being leveraged to identify the predictors associated with prolonged hospital stays. Essentially, these statistical algorithms are capable of learning patterns from nonlinear, high-dimensional data, while simultaneously considering the interactions between multiple relevant variables. This research aims to develop and implement advanced machine learning (ML) models to predict which patients may experience prolonged hospitalization following surgery. Our methodology seeks to address limitations present in conventional prediction models through the incorporation of more sophisticated and advanced ML techniques. In addition, we aim to deploy our predictive tool as an online resource accessible to all interested parties free of charge.

## Methods

### Cohort and study design

The Ethics Committee of the Hospital granted approval for this retrospective cohort study, in which researchers examined the medical files of 580 individuals diagnosed with tuberculous spondylitis. The study specifically targeted individuals who underwent posterior decompression and instrumentation surgery at our medical center during the period from January 1, 2016, to December 31, 2022. To adhere to best practices and current regulatory legislation for data privacy, a thorough de-identification procedure was carried out prior to conducting any data analysis. The diagnoses of tuberculosis spondylitis (TS) relied upon meticulous clinical evaluations, laboratory findings, and radiographic evidence such as X-ray, computed tomography, and magnetic resonance imaging. Postoperative confirmation of TS diagnoses was accomplished through histopathological examination and/or the detection of acid-fast bacilli using *Ziehl–Neelsen* staining, and/or the growth of *Mycobacterium* tuberculosis in culture specimens obtained from bone marrow, abscesses, or tissue [14]. Inclusion criteria were: (1) age ≥ 18 years; (2) patients present with a range of symptoms, including but not limited to continuous or unremitting local pain in the neck or back, fever, chills, weight loss, malaise, neurological symptoms, or limited range of motion in the spine; (3) complete information of lab test; (4) complete records of medical imaging examination consisting of preoperative imaging modalities including X-ray, computed tomography (CT), and magnetic resonance imaging (MRI) were performed prior to the initiation of surgical therapy; (5) confirmation the diagnosis using the gold standard (microbiology/histopathology examination); and (6) we conducted a surgical procedure involving posterior lumbar spine decompression, debridement, and stabilization in the affected area. To mitigate potential confounding variables associated with multiple procedures, we included only one surgical intervention for each patient who may have undergone several eligible spine operations. More specifically, this study encompassed patients who underwent the procedure of posterior lumbar spine decompression, debridement, and stabilization in the lesion region.

Patients who fulfilled any of the following criteria were not included in the study: (1) age < 18 years; (2) received conservative treatment; (3) complicated with tumors; (4) complicated with disc herniation or spondylolisthesis; (5) complicated with spine fracture; (6) severe chronic internal medicine disease; (7) scoliosis and Kyphosis; (8) minimally invasive fusion surgery; and (9) patients with more than 30% missing data were excluded.

### Candidate variables in the models

This study utilized a comprehensive range of five distinct categories of features commonly found in electronic health records (EHRs). These categories encompassed: (1) demographic information such as age, sex, and marital status; (2) patient symptoms and medical history; (3) preoperative laboratory data and imaging features derived from X-rays, computed tomography (CT) scans, and magnetic resonance imaging (MRI); (4) surgical factors including operation duration, intraoperative blood loss, and the need for transfusions; and (5) postoperative details such as the extent of drainage from the surgical site. All images used in this study underwent review and analysis by a chief physician who was unaware of the clinical and laboratory results. It is worth noting that an expert physician meticulously evaluated all images used in this study, with no knowledge of the clinical and laboratory findings. Since patients often undergo multiple biological and imaging tests throughout their treatment, the data used for the prediction models were based on the admission data, representing the initial test value or report for each specific test.

### Target outcomes class imbalance

**Fig. 1:**
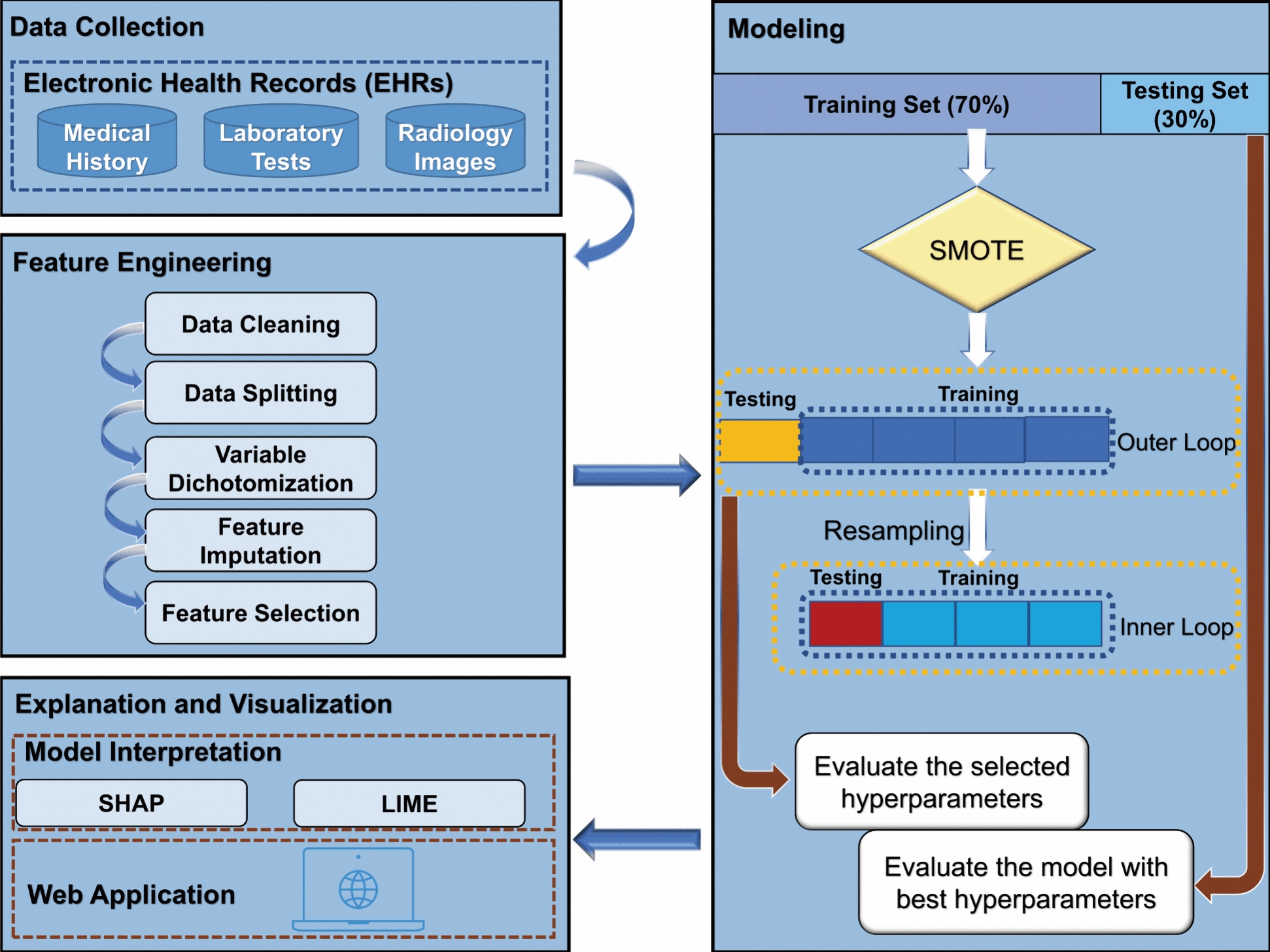
Pipelines for the whole research

The primary objective of this study was to analyze the duration of hospital stays (referred to as length of stay or LOS) among participants who underwent surgery. Specifically, it aimed to examine hospital stays beyond the 75th quartile of LOS. A total of 580 participants were included in the study, with 127 demonstrating a normal LOS. This substantial difference in sample size resulted in an imbalance between participants with normal and prolonged LOS. To address this issue, the Synthetic Minority Oversampling Technique (SMOTE) was employed to oversample the minority class, namely, the group with prolonged LOS, in the training set [15, 16] (Fig. 1). To ensure the robustness and generalizability of each model, both feature selection and data balancing processes were limited to the training set.

SMOTE sampling method is a popular technique utilized for resampling data. The approach involves selecting a limited number of samples as the basis for generating an augmented dataset. From this pool of selected samples, new samples are created by randomly selecting auxiliary samples from k neighboring samples of the same category, based on sample multiplicity [17]. The process is then repeated n times to achieve a balanced and representative dataset that minimizes over-fitting [18].

### Data pre-processing

Prior to data separation, the data set was divided into training (70%) and test (30%) sets. The stratification of the data sets was based on the outcome variable, ensuring that the proportions of positive and negative samples, specifically normal and prolonged length of stay, remained consistent between the training and testing sets, as well as the overall sample.

The transformation of raw electronic health record (EHR) data into machine-learning suitable variables is a noteworthy aspect. Pain levels were categorized into two groups, namely, moderate (VAS ≤ 5) and severe (VAS > 5), using the visual analog scale (VAS). In addition, during the patient’s initial visit, the recorded fever grade was classified as either low (< 38.5 °C) or high (≥ 38.5 °C). Furthermore, the definition of severe vertebral destruction encompassed damage affecting one-third or more of the vertebrae.

Following data separation, missing variable imputation and normalization were separately conducted on both the training and testing sets to avoid data leakage [19]. To normalize the numeric variables, the *z*-score method was applied. This involved subtracting the mean value of the sample from each individual value and then dividing the difference by the sample standard deviation. To standardize the features, the standard scaler was fitted to the training set by removing the mean and scaling to unit variance. Subsequently, the same scaler was applied to transform the data in both the training and test sets.

The input parameter values and external model configurations, known as hyperparameters, were defined using the training set to optimize predictive accuracy. Conversely, the test set consisted of new data that was not used during algorithm training, enabling the evaluation of model performance on previously unseen data. To impute missing variables, we employed the multiple imputations by chained equations (MICE) technique [20].

### Predictors selection

Given the potential challenges posed by noisy, redundant, and irrelevant variables in hindering the performance of learning algorithms, it is imperative to employ effective feature selection techniques. In this study, we employed several methods such as variance thresholding, supper vector machine classification (SVC)-based recursive feature elimination (SVC–RFE), Pearson correlation analysis, LASSO, and permutation importance ranking to address these challenges. Specifically, we utilized the Pearson correlation analysis to eliminate highly correlated variables, while variables with Pearson correlation coefficients above 0.90 were considered to exhibit low quality and high redundancy and were therefore excluded from the analysis altogether. The LASSO method, a supervised machine-learning technique and a penalized regression model capable of enhancing prediction accuracy and interpretability of the statistical model, was used to select the most useful predictive features in high-dimensional variables [21]. By means of discarding confounding variables and minimizing the coefficients of comparatively irrelevant variables to 0 using a tenfold cross-validation technique in the training data set, we were able to construct a statistically significant model with superior performance metrics.

Recursive feature elimination (RFE) is a technique used to select features by iteratively assessing smaller and smaller subsets based on weighted assignments from an external estimator [22]. A defined property or callable function is used to determine the relevance of each feature after training the estimator with a starting set of characteristics. The least important characteristics in the present collection are then removed. The trimmed set is then subjected to this recursive approach again until the desired number of chosen features is obtained. In this study, the least significant characteristics were determined using the SVC–RFE approach and fivefold cross-validation [23].

Automated methods for choosing pertinent characteristics are used in machine learning (ML), some of which are based on built-in measurements of varying relevance. The Gini Importance (MDI: Mean Decrease in Impurity) and the Permutation Importance (MDA: Mean Decrease in Accuracy) are two often used metrics [24, 25]. These measures rank the relevance of features concerning their utility in predicting outcomes. The importance of features is defined as their contribution towards improving the overall predictive performance of the model. The *Gini* Importance index is an example of a measure that can be derived from the Gini index, which is used to assess node impurity. Nonetheless, relying on the Gini Importance index to evaluate feature relevance may lead to biased results due to its use of the Gini-gain splitting criterion, which can be influenced by selection bias when analyzing factorial data [26].

*Breiman* first proposed Permutation feature importance metrics for feature selection in Random Forests, which were later refined by *Altmann* to address potential biases associated with the *Gini* Importance and entropy criterion [27]. Consequently, Permutation variable importance has emerged as a more reliable approach for selecting features, particularly when working with continuous and highly categorical variables [28]. The Permutation variable importance score is calculated using two widely used ensemble tree-based machine learning algorithms—random forest (RF) and XGBoost in this research. Features with higher Permutation variable importance scores are generally more strongly related to predicting prolonged length of stay (LOS).

To improve the efficiency of our feature selection process, we generated an intersection of the feature datasets based on the aforementioned methods. The resulting set of selected features was then employed during the machine learning training phase.

### Candidate model frameworks and hyper-parameters tuning

In this study, several algorithms were explored for classification tasks. Naive Bayes (NB) is a supervised probabilistic machine learning algorithm that relies on Bayes’ Theorem and assumes strong independence between input features [29]. Logistic regression (LR) is a predictive machine learning algorithm with a complex cost function and does not assume feature independence [30, 31]. *K*-Nearest Neighbor is a popular algorithm for pattern recognition that predicts the new query instance’s category based on the majority of *k*-nearest neighbor categories [32]. Random forest is an ensemble learning method that addresses the issue of overfitting in decision trees by constructing multiple decision trees during the training process [33]. Support vector machines is a powerful machine learning algorithm that learns well with small parameters and is robust against various model violations [34]. A decision tree is a hierarchical framework that splits independent variables recursively into homogeneous zones [35]. XGBoost is an optimized distributed gradient-boosting system that has been specifically designed for high efficiency, flexibility, and portability [36]. LightGBM is a tree-based machine learning algorithm that utilizes gradient boosting learning and histogram-based algorithms and grows the tree leafwise [37].

To enhance the predictive accuracy of certain categories while limiting the incidence of false positives, our research team conducted a thorough investigation into the collective efficacy of various machine learning models. Our primary objective was to generate optimal predictions that would yield the best possible outcomes. To accomplish this goal, we implemented a technique known as the voting model or majority voting ensemble, in which we combined the results generated by multiple machine learning algorithms. The purpose of this approach was to identify the most prevalent prediction, which would then serve as the final outcome. The voting model approach is particularly beneficial in cases where multiple models perform well at predicting certain categories, a scenario that was observed in our research. By utilizing this technique, we aimed to achieve superior performance compared to individual models. The voting model approach encompasses two methods: hard voting and soft voting. Hard voting involves combining the predictions for each label and selecting the one with the highest number of votes. In contrast, soft voting involves summing up the probabilities assigned to each class and selecting the prediction with the highest probability. In our research, we utilized the soft-voting method to ensure the model's consistent contributions across all categories.

The investigation comprised a training procedure that made use of grid-search and layered cross-validation. An outer loop for selecting the test set in each fold and an inner loop for further segmenting the data into training and validation sets made up the nested cross-validation technique. The dataset was first divided into train and test sets for the outer loop. Grid-search was used within the inner loop to do *k*-fold cross-validation on the train set to find the ideal set of hyperparameters from a predetermined list [38]. For each optimization trial, the data was divided into training and validation sets, followed by the implementation of inner *k*-fold cross-validation. The aim was to maximize the overall accuracy from the *k*-fold validation, to determine the optimal combination of hyperparameters. This optimal combination was then used to train the final model using all available training samples. Subsequently, the resulting model was tested on the outer fold of the test set. This entire procedure was repeated for all outer folds, and the evaluation metrics were computed based on the aggregated test results (Fig. 1).

### Model performance assessment and comparison

Evaluation and comparison of the predictive model's performance are crucial in the modeling process after constructing and training the model. For this study, various standard performance indicators were utilized to assess the classifiers’ performance. These indicators include accuracy, specificity (also known as recall), sensitivity, negative predictive value (N.P.V.) (also known as precision), positive predictive value (P.P.V.), area under the receiver-operating characteristic curve (AUC), F1-Score, Log-Loss, and Brier-Score. To evaluate calibration, the Brier score was used, with scores ranging from 0 to 1, where lower scores indicate better calibration. Discrimination was measured by the area under the receiver operating characteristic curve (ROC–AUC). A value of 0.50 is used as a reference for a random estimator, while a value of 1.0 is used as a reference for a model with perfect discriminant ability. These metrics were computed using the binary confusion matrix described below.

In our study, patients with prolonged length of stay (LOS) were classified as having prolonged LOS if correctly detected as true positive (TP), while those with normal LOS were classified as true negative (TN). On the other hand, false positive (FP) occurred when a patient with normal LOS was falsely detected as having prolonged LOS, and false negative (FN) was when a patient with prolonged LOS was incorrectly categorized as having normal LOS. We evaluated the accuracy of the predictive models by computing performance evaluation matrices involving TP, TN, FP, and FN classifications for both prolonged and normal LOS outcomes.

AUC and Brier score, which were kept for additional study, were the key criteria used to choose the best model. During the training stage, we assessed the ROC–AUC values for each stage of the fivefold cross-validation method. We also showed the calibration plot and the decision curve analysis curves to help you assess the performance of the model you choose.

### Model explanation

Feature selection techniques that estimate the relevance of variables are limited in their ability to describe patterns of dependency between features and responses. Instead of providing a comprehensive understanding, they merely convey the intensity of this dependency as a single number, making the resulting findings difficult to interpret. To overcome this limitation, we utilized two procedures, namely, Shapley additive explanations (SHAP) and local interpretable model-agnostic explanations (LIME), to explain the selected model and assess the contribution of each variable to its performance. These procedures provide valuable insights into the predictive importance of each variable and the direction of their values [12, 39] (Fig. 1).

The SHAP approach can make it easier to comprehend the chosen model. Important characteristics in the model are those with a high SHAP value, whereas those with a low SHAP value have a detrimental influence on the model’s output [40]. On the other hand, characteristics with a positive value support the desired result (prolonged LOS). To illustrate the significance and direction of each predictive predictor, plots were created. Each predictive variable’s location on the *y*-axis was arranged according to its relative relevance, with the most significant predictors at the top. The location of each point on the *x*-axis for each predictive variable signified the contribution of each participant to the total value of the Shapley additive explanations, with significant positive contributions on the far right (red signifying greater values or the existence of binary components).

To explain model predictions, the local interpretable model-agnostic explanation (LIME) technique is used. The rationale behind this approach is based on the use of a local linear approximation of the model behavior to predict a single sample, which can be more confidently trusted. LIME is a widely recognized tool proposed by Ribeiro et al. [41] that specifically aims to help explain the decision-making processes of complex black-box models. LIME is used to create a new dataset by randomly disrupting the samples, which is then used to train a linear model that locally approximates the black-box model [13]. In doing so, LIME enables us to obtain insights into the local decision-making behaviors of the black-box model using an interpretable model.

### Web application deployment

The final model was deployed as a web application. With the growing number of scientific findings emerging from omics studies, there is a demand for novel translational medicine applications and bioinformatics tools. These tools aim to facilitate the integration of these findings into clinical practice, bridging the gap between laboratory research and patient care. The translation of knowledge from bench to bedside not only encourages the development of new biotechnological products but also contributes to improving patients’ health [42].

### Statistical analysis

When comparing categorical variables, the appropriate Chi-square or Fisher’s exact test was used to present the data as numbers and percentages. Continuous variables that met the criterion for normal distribution were described as mean values accompanied by their standard deviations [mean (SD)], and compared using the two-tailed Student’s *t* test. Alternatively, median values along with their interquartile ranges [median (IQR)] and *Wilcoxon–Mann–Whitney U* test were applied to continuous variables that did not meet this normality assumption. Statistical significance was defined as a two-sided *P* value of 0.05. Graphs presented in this article were constructed using Microsoft Excel, R Studio 4.1.2 (R Development Core Team), and Python version 3.9.7.

## Results

### Baseline characteristics

The data set comprises 580 participants, out of which 21.9% (127) experienced a prolonged length of stay (LOS). Among the participants, 403 were men (70.2%) and 173 were women (29.8%). The participants were 47.2 years old on average. Twenty-two percent, or 117 people, had a history of TB. Older age was associated with prolonged LOS among the patients in the TS group. Further details can be found in Table 1. The proportion of minority samples oversampled by the SMOTE algorithm can be found in supplementary files.

**Table 1:** Baseline characteristics of patients

Variables	ALL (*N* = 580)	Training set (*N* = 412)	Testing set (*N* = 168)	*P*
LOS				0.704
Normal	453 (78.1%)	324 (78.6%)	129 (76.8%)	
Prolonged	127 (21.9%)	88 (21.4%)	39 (23.2%)	
Age	47.2 ± 19.7	47.5 ± 20.0	46.6 ± 19.1	0.603
Gender				0.201
Female	173 (29.8%)	116 (28.2%)	57 (33.9%)	
Male	407 (70.2%)	296 (71.8%)	111 (66.1%)	
BMI (Kg/m^2^)	22.8 ± 5.24	23.0 ± 5.69	22.3 ± 3.93	0.111
Fever				0.183
High	81 (14.0%)	52 (12.6%)	29 (17.3%)	
Low	499 (86.0%)	360 (87.4%)	139 (82.7%)	
Pain				0.307
Moderate	307 (52.9%)	212 (51.5%)	95 (56.5%)	
Severe	273 (47.1%)	200 (48.5%)	73 (43.5%)	
Wasting				0.443
No	361 (62.2%)	261 (63.3%)	100 (59.5%)	
Yes	219 (37.8%)	151 (36.7%)	68 (40.5%)	
Past history of tuberculosis				0.713
No	463 (79.8%)	331 (80.3%)	132 (78.6%)	
Yes	117 (20.2%)	81 (19.7%)	36 (21.4%)	
WBC (10^9^/L)	6.87 ± 2.38	6.87 ± 2.23	6.88 ± 2.71	0.969
ESR (mm/h)	43.1 ± 19.0	42.5 ± 18.5	44.7 ± 19.9	0.222
CRP (mg/L)	36.5 ± 32.9	37.9 ± 33.7	33.2 ± 30.6	0.101
HB (g/L)	120 ± 21.0	120 ± 20.9	119 ± 21.1	0.508
TG (mmol/L or mg/dL)	1.21 ± 0.59	1.21 ± 0.55	1.19 ± 0.67	0.722
TC (mmol/L or mg/dL)	3.86 ± 0.93	3.86 ± 0.93	3.86 ± 0.92	0.984
HDL (mmol/L or mg/dL)	1.08 ± 1.39	1.02 ± 0.31	1.23 ± 2.54	0.294
LDL (mmol/L or mg/dL)	2.64 ± 0.78	2.64 ± 0.79	2.65 ± 0.76	0.841
ALB (g/L)	38.0 ± 5.47	37.9 ± 5.52	38.4 ± 5.36	0.284
AST (U/L)	24.4 ± 27.8	23.7 ± 24.0	26.3 ± 35.4	0.383
ALT (U/L)	24.3 ± 33.6	23.4 ± 30.8	26.4 ± 39.7	0.373
GGT (U/L)	51.0 ± 51.1	52.4 ± 54.3	47.6 ± 42.1	0.250
ALP (U/L)	105 ± 45.0	106 ± 47.2	102 ± 38.9	0.291
MRI vertebral destruction				0.306
Moderate	62 (10.7%)	48 (11.7%)	14 (8.33%)	
Severe	518 (89.3%)	364 (88.3%)	154 (91.7%)	
MRI spinal stenosis				0.537
No	248 (42.8%)	180 (43.7%)	68 (40.5%)	
Yes	332 (57.2%)	232 (56.3%)	100 (59.5%)	
MRI paravertebral abscess				0.461
No	120 (20.7%)	89 (21.6%)	31 (18.5%)	
Yes	460 (79.3%)	323 (78.4%)	137 (81.5%)	
MRI psoas abscess				0.822
No	358 (61.7%)	256 (62.1%)	102 (60.7%)	
Yes	222 (38.3%)	156 (37.9%)	66 (39.3%)	
MRI epidural abscess				0.614
No	438 (75.5%)	314 (76.2%)	124 (73.8%)	
Yes	142 (24.5%)	98 (23.8%)	44 (26.2%)	
CT marginal osteophyte				1.000
No	113 (19.5%)	80 (19.4%)	33 (19.6%)	
Yes	467 (80.5%)	332 (80.6%)	135 (80.4%)	
CT endplate sclerosis				0.968
No	184 (31.7%)	130 (31.6%)	54 (32.1%)	
Yes	396 (68.3%)	282 (68.4%)	114 (67.9%)	
CT vertebral destruction				0.363
Moderate	155 (26.7%)	115 (27.9%)	40 (23.8%)	
Severe	425 (73.3%)	297 (72.1%)	128 (76.2%)	
CT spinal stenosis				0.463
No	271 (46.7%)	197 (47.8%)	74 (44.0%)	
Yes	309 (53.3%)	215 (52.2%)	94 (56.0%)	
CT paravertebral abscess				0.089
No	158 (27.2%)	121 (29.4%)	37 (22.0%)	
Yes	422 (72.8%)	291 (70.6%)	131 (78.0%)	
CT epidural abscess				0.639
No	373 (64.3%)	262 (63.6%)	111 (66.1%)	
Yes	207 (35.7%)	150 (36.4%)	57 (33.9%)	
X-ray disc height loss				0.849
No	129 (22.2%)	93 (22.6%)	36 (21.4%)	
Yes	451 (77.8%)	319 (77.4%)	132 (78.6%)	
X-ray vertebral destruction				0.024
No	138 (23.8%)	109 (26.5%)	29 (17.3%)	
Yes	442 (76.2%)	303 (73.5%)	139 (82.7%)	
X ray endplate sclerosis				0.298
No	249 (42.9%)	183 (44.4%)	66 (39.3%)	
Yes	331 (57.1%)	229 (55.6%)	102 (60.7%)	
X ray osteophyte				0.018
No	260 (44.8%)	198 (48.1%)	62 (36.9%)	
Yes	320 (55.2%)	214 (51.9%)	106 (63.1%)	
X ray bone bridge				0.796
No	475 (81.9%)	339 (82.3%)	136 (81.0%)	
Yes	105 (18.1%)	73 (17.7%)	32 (19.0%)	
Day				0.689
Friday	96 (16.6%)	70 (17.0%)	26 (15.5%)	
Monday	107 (18.4%)	81 (19.7%)	26 (15.5%)	
Thursday	94 (16.2%)	63 (15.3%)	31 (18.5%)	
Tuesday	133 (22.9%)	94 (22.8%)	39 (23.2%)	
Wednesday	150 (25.9%)	104 (25.2%)	46 (27.4%)	
Surg time (min)				0.948
< 202	393 (67.8%)	280 (68.0%)	113 (67.3%)	
≥ 202	187 (32.2%)	132 (32.0%)	55 (32.7%)	
Drainage (mL)				0.098
< 290	449 (77.4%)	327 (79.4%)	122 (72.6%)	
≥ 290	131 (22.6%)	85 (20.6%)	46 (27.4%)	
Multiple sections				0.982
No	388 (66.9%)	275 (66.7%)	113 (67.3%)	
Yes	192 (33.1%)	137 (33.3%)	55 (32.7%)	
Segment (s)	2.79 ± 1.34	2.78 ± 1.36	2.80 ± 1.31	0.872
Infusion volume (mL)				0.832
< 1675	399 (68.8%)	285 (69.2%)	114 (67.9%)	
≥ 1675	181 (31.2%)	127 (30.8%)	54 (32.1%)	
Blood loss (mL)				0.311
< 235	345 (59.5%)	251 (60.9%)	94 (56.0%)	
≥ 235	235 (40.5%)	161 (39.1%)	74 (44.0%)	
Transfusions				0.229
No	507 (87.4%)	365 (88.6%)	142 (84.5%)	
Yes	73 (12.6%)	47 (11.4%)	26 (15.5%)	

LOS (length of stay) refers to hospitalization time (day) after surgery; BMI (Body mass index) Kg/m^2^; WBC (White Blood Cell Count): 10^9^/L; ESR (Erythrocyte Sedimentation Rate): mm/hr; CRP (C-Reactive Protein): mg/L; HB (Hemoglobin): g/L; TG (Triglycerides): mmol/L or mg/dL; TC (Total Cholesterol): mmol/L or mg/dL; HDL (High-Density Lipoprotein Cholesterol): mmol/L or mg/dL; LDL (Low-Density Lipoprotein Cholesterol): mmol/L or mg/dL; ALB (Albumin): g/L; AST (Aspartate Aminotransferase): U/L; ALT (Alanine Aminotransferase): U/L; GGT (Gamma-Glutamyl Transferase): U/L; ALP (Alkaline Phosphatase): U/L

### Feature selection

**Fig. 2:**
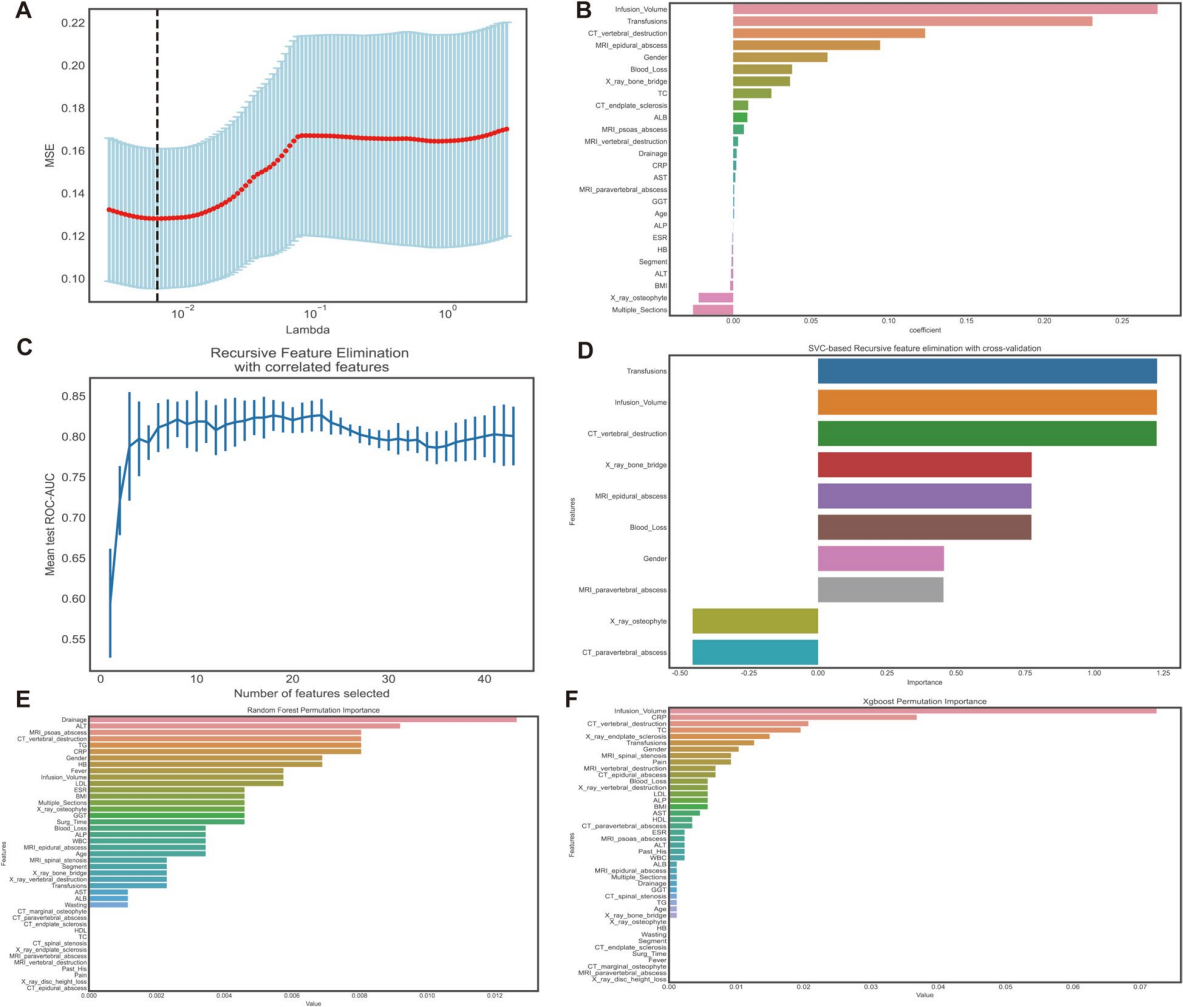
Dimension reduction. **A**, **B** LASSO method; **C** support Vector Machine Classifier based recursive features elimination (SVC–RFE) with tenfold cross-validation to confirm a minimal number of important features; **D** selected features with SVC–RFE; **E** random forest permutation importance ranking; **F** XGBoost permutation importance ranking

As superfluous information may leave the classifications of LOS unsatisfactory, based on the LASSO method, 26 meaningful variables were filtered out, as shown in Fig. 2A, B. Furthermore, permutation importance calculated by fitting RF and XGBoost algorithms was also taken into consideration (Fig. 2C, D). Firstly, we performed SVC–RFE with fivefold cross-validation to determine the optimal subset of relevant features with minimal redundancy (Fig. 2E). Upon analyzing the plot, it becomes evident that the increase in the number of features beyond 10 does not result in significant elevation. Therefore, we selected ten variables as the most suitable and pertinent number, ensuring the inclusion of only the most relevant variables (Fig. 2F). Regarding the clinical practice and clinical relevance, and combining the results of the above screening procedure. The final training and testing dataset was composed of 11 variables, which are CRP, transfusions, infusion volume, blood loss, X-ray bone bridge, X-ray osteophyte, CT-vertebral destruction, CT-paravertebral abscess, MRI-paravertebral abscess, MRI-epidural abscess, postoperative drainage.

**Fig. 3:**
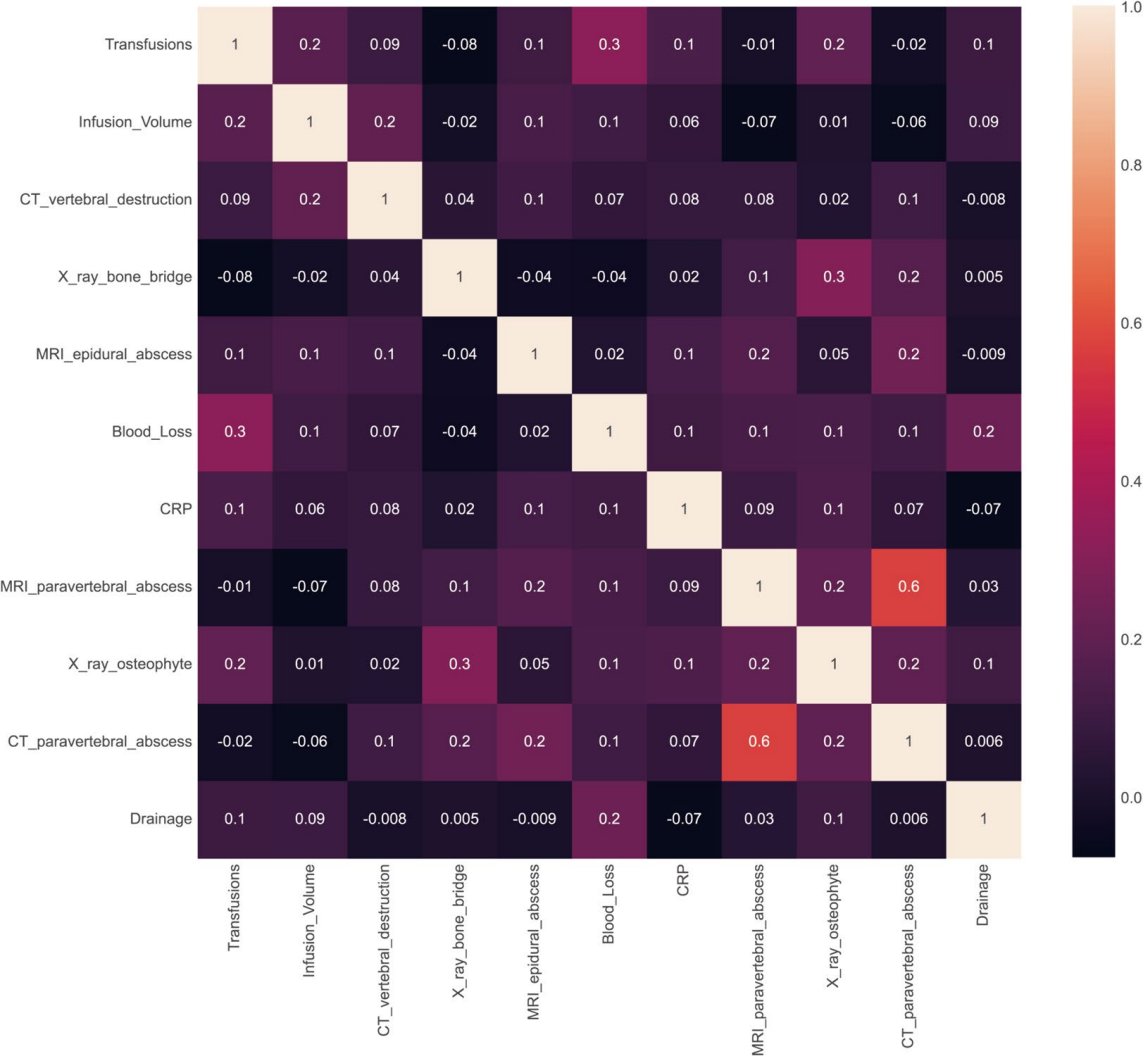
Heatmap of the correlation coefficient of features

To alleviate the issue of variable collinearity, we also examined the correlation between the variables in the dataset using three feature selection methods. First, we generated a heatmap to visualize the correlation coefficients and associated *P* values of the variables. As depicted in Fig. 3, all the coefficients were less than 0.6, although some variables exhibited statistical significance (*P* < 0.05). This implies that there is some correlation between certain variables, but the level of correlation is weak (indicated by small correlation coefficients, i.e., less than 0.6).

### Model establishment and evaluation

**Fig. 4:**
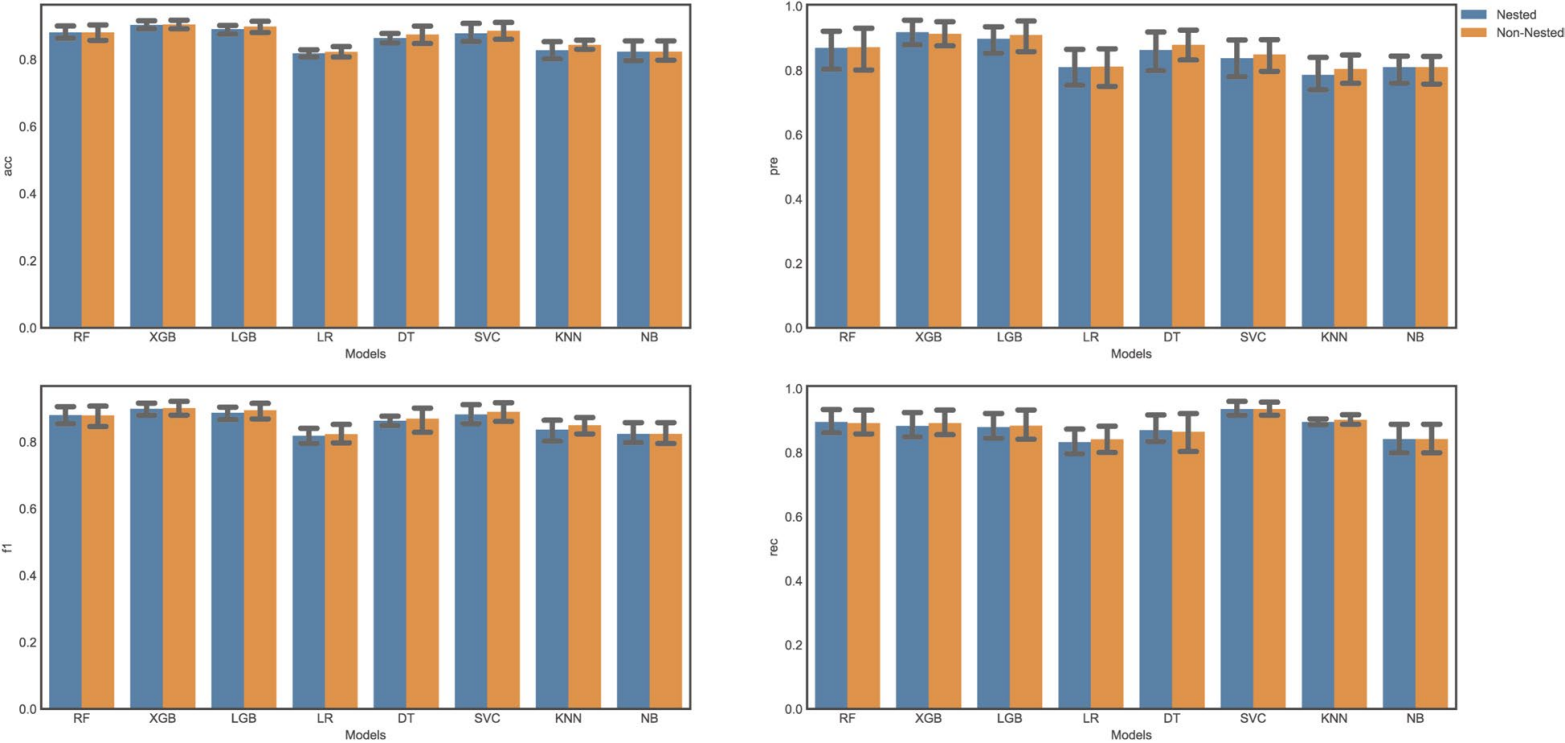
Bar plot of comparison of nested and non-nested cross-validation approach

**Fig. 5:**
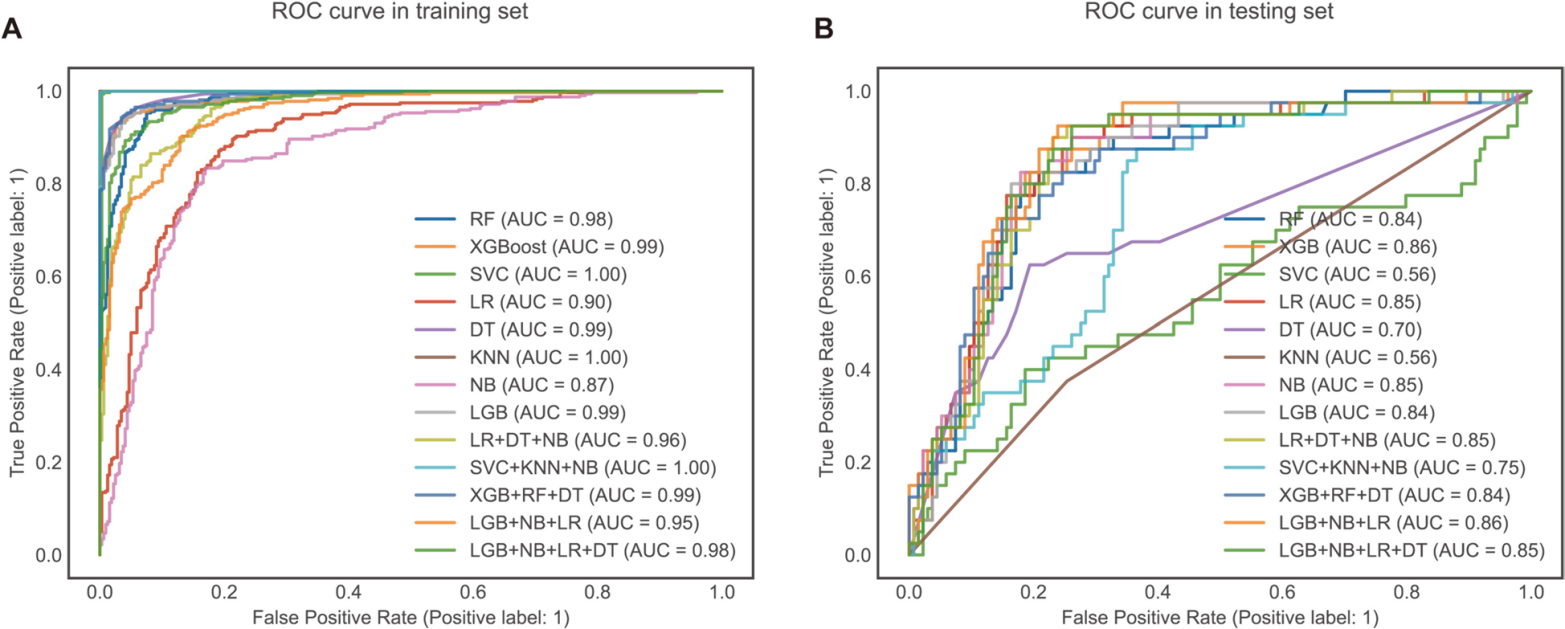
Each model’s Receiver Operating Characteristic (ROC). Receiver operating characteristic curve, which illustrates the tradeoff between the model’s sensitivity and false positive rate in light of the range of expected probabilities of prolonged PLOS. The area under the curve shows the model’s ability to predict whether prolonged PLOS would develop or not. A random estimator is referenced using a value of 0.5 (shown by the dotted line), whereas a perfect model would display a value of 1.0

As illustrated in Fig. 4, we compared both nested and non-nested cross-validation approaches when training our models. It can be discerned that the non-nested method will lead to an over-optimistic performance-assessment value than the nested method, which has prone to over-fitting. Therefore, nested-cross validation was adopted in our research. All classification models obtained well discrimination ability. The machine learning models were evaluated individually, with Random Forest (AUC = 0.84, Brier Score = 0.143), XGBoost (AUC = 0.86, Brier Score = 0.126), Support Vector Classifier (AUC = 0.56, Brier Score = 0.223), Logistic Regression (AUC = 0.85, Brier Score = 0.155), Decision Tree (AUC = 0.65, Brier Score = 0.205), k-Nearest Neighbors (AUC = 0.56, Brier Score = 0.339), Naive Bayes (AUC = 0.85, Brier Score = 0.176), and LightGBM (AUC = 0.84, Brier Score = 0.135) achieving varying scores. Ensemble models using soft voting techniques were also considered, including LR + DT + NB (AUC = 0.85, Brier Score = 0.145), SVC + KNN + NB (AUC = 0.75, Brier Score = 0.183), XGB + RF + DT (AUC = 0.83, Brier Score = 0.14), LGB + NB + LR (AUC = 0.86, Brier Score = 0.139), and LGB + NB + LR + DT (AUC = 0.84, Brier Score = 0.139) (Fig. 5). What is more, we also calculated other metrics to measure the comprehensive capability of each algorithm. Brier score and log loss were given more attention. The Brier score was used to measure the accuracy of the predictions using the predicted probabilities in their continuous form; the model with the lowest Brier Score is considered to have the greatest predictive performance. Compared with other models, the XGBoost model achieved the best performance with the highest AUC of 0.86 and the lowest Brier score of 0.126 in the testing group. More detailed information about each model’s results can be found in in Table 2. Thus, the XGBoost model was maintained for further analysis, interpretation, and visualization. The prediction results of selected model by different groups can be found in supplementary files.

**Table 2:** Evaluation of the classification algorithms

Classifiers	Brier loss	Log loss	Acc	F1 score	Sen	Spe	Npv	Ppv
RF	0.143	0.439	0.787	0.554	0.575	0.851	0.870	0.535
XGB	0.126	0.389	0.799	0.521	0.475	0.896	0.851	0.576
LGB	0.135	0.415	0.799	0.545	0.525	0.881	0.861	0.568
LR	0.155	0.495	0.799	0.646	0.800	0.799	0.930	0.542
SVC	0.223	0.924	0.713	0.342	0.325	0.828	0.804	0.361
KNN	0.339	11.712	0.661	0.337	0.375	0.746	0.800	0.306
NB	0.176	0.776	0.787	0.648	0.850	0.769	0.945	0.523
DT	0.205	2.784	0.736	0.410	0.400	0.836	0.824	0.421
Ensemble mode1 1	0.145	0.453	0.805	0.646	0.775	0.813	0.924	0.554
Ensemble mode1 2	0.183	0.601	0.718	0.380	0.375	0.821	0.815	0.385
Ensemble mode1 3	0.139	0.424	0.782	0.513	0.500	0.866	0.853	0.526
Ensemble mode1 4	0.140	0.442	0.799	0.639	0.775	0.806	0.923	0.544
Ensemble mode1 5	0.144	0.449	0.793	0.609	0.700	0.821	0.902	0.538

Model 1: LR + LR + NB, Model 2: SVC + KNN + NB, Model 3: XGB + RF + DT, Model 4: LGB + NB + LR, Model 5: LGB + NB + LR + DT

**Fig. 6:**
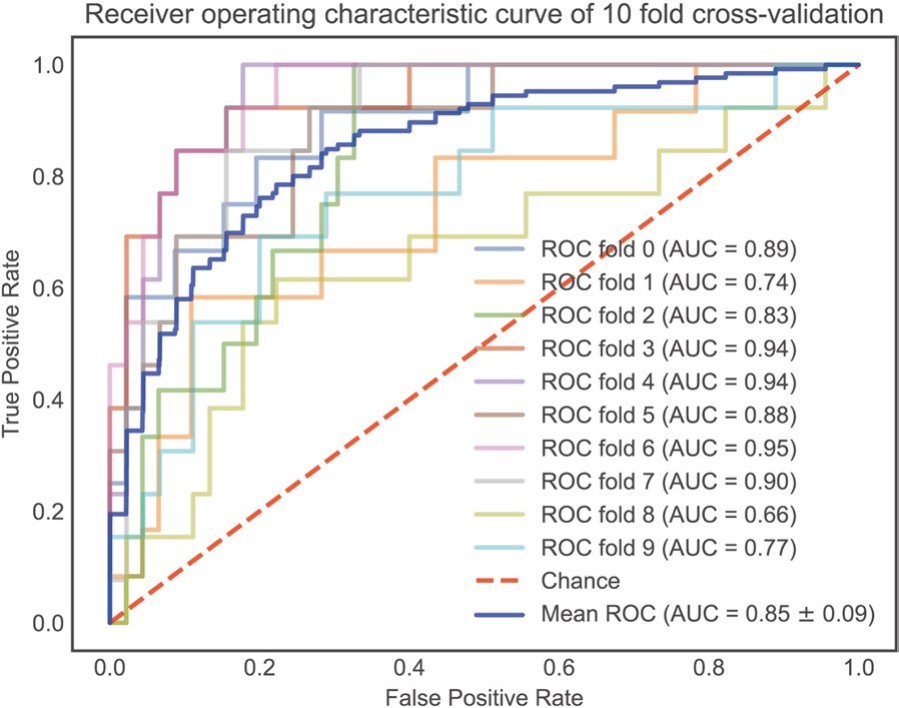
ROC with tenfold cross-validation. Receiver operating characteristic curve, illustrating the model’s trade-off between sensitivity and the rate of false positives in light of the range of expected probabilities of future prolonged PLOS. The area under the curve shows how well the model can predict whether future prolonged PLOS will develop or not. A random estimator’s reference value is 0.5, while the value of a perfect model would be 1.0 (shown by the dotted line)

**Fig. 7:**
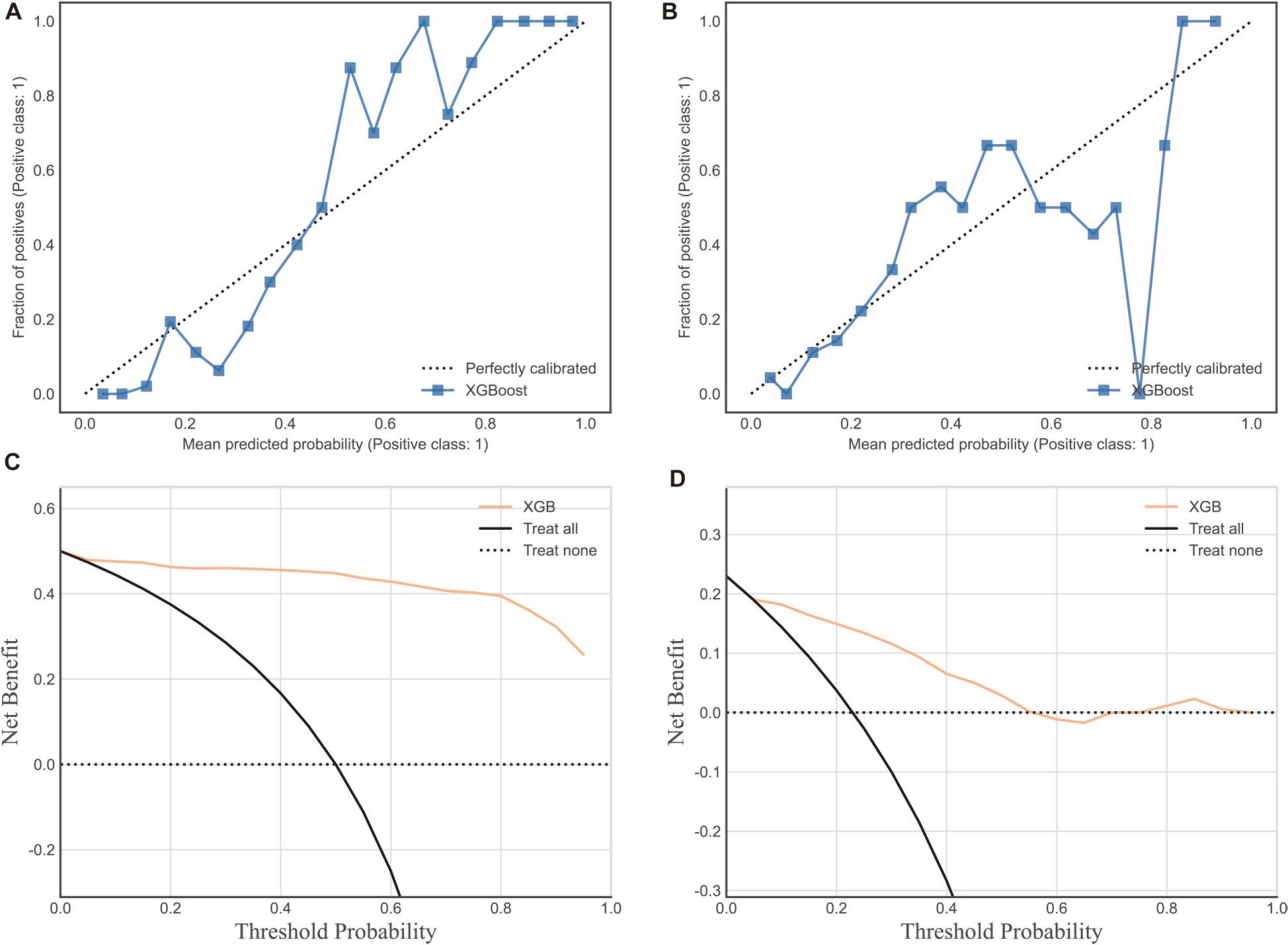
Calibration plots were generated for both the training set (**A**) and test set (**B**). These plots illustrate the disparity between the model’s predicted probability of prolonged length of stay after surgery and the actual frequency observed in the study sample. The dashed line represents the ideal calibration of the model. The results indicate a close resemblance between the predicted and observed probabilities. Decision curve analysis curves were plotted for both the training set (**C**) and the testing set (**B**). Positioned in the upper right corner, the decision curve analysis curve clearly demonstrates the considerable net benefits offered by the predictive model

To further explore the generic discrimination ability of our model. It was evaluated using twofold cross-validation, where it displayed mean AUC was 0.85 ± 0.09 which is almost equal to the AUC value in the validation set (Fig. 6). We also examined the clinical practical usefulness of the XGBoost model via calibration plot and decision analysis curve (Fig. 7).

### Model interpretation of selected predictors

**Fig. 8:**
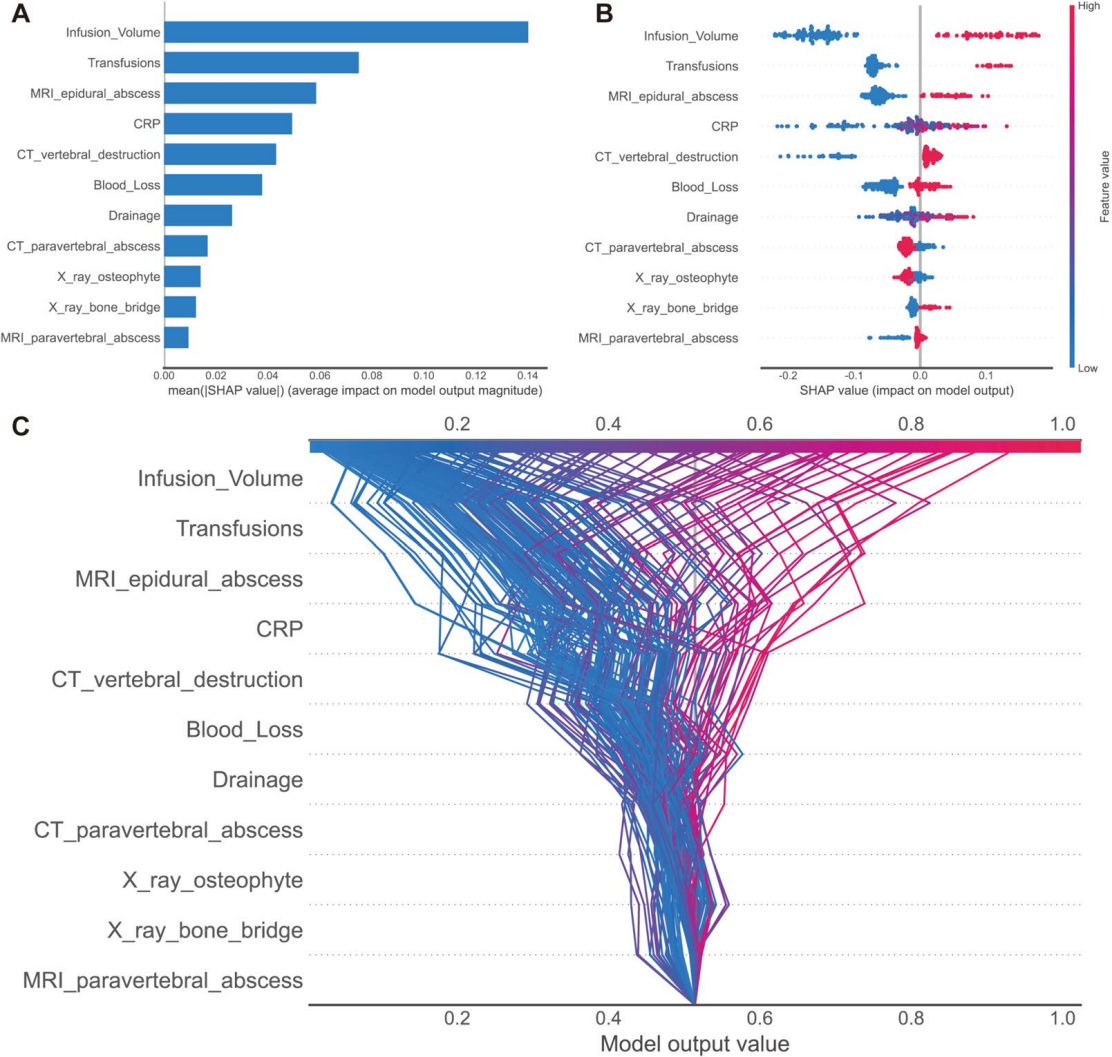
A graphic representation of the impact of predictors on the outcome is depicted using the SHapley Additive exPlanations (SHAP) technique. Firstly, a SHAP bar plot (**A**) shows the features in descending order from top to bottom, with the most influential risk factors at the top. Secondly, a SHAP summary plot (**B**) presents lines representing predictors, arranged in decreasing order of importance for outcome prediction. The dots on each line represent the SHAP values corresponding to the predictors at each index checkup visit. The color and position of the dots indicate the strength and direction of the association. Specifically, red denotes a higher value (or presence of a specific category for categorical variables), while blue signifies a lower value (or absence of the category). In addition, dots on the left side of the chart indicate a decreased risk of the outcome, with greater leftward displacement indicating a higher impact on risk reduction. Conversely, dots on the right side represent an increased risk, with greater rightward displacement indicating a stronger impact on risk elevation. Lastly, a SHAP decision plot (**C**) provides an overview of how each critical parameter influences the final decision. Each colored line in the figure represents the predicted outcome for an individual patient

As data are entered and choices are made without a clear grasp of the process between them, machine learning models are frequently criticized for being opaque. To solve this problem, we used the Shapley additive explanations (SHAP) technique, which makes use of SHAP values to evaluate the interpretability of the model by providing a visual representation of the impact of each chosen predictor. The purpose of Fig. 8A, which represents the likelihood of extended LOS, is to evaluate the unique influence of each predictor by the size of its value (coded by a gradient of colors) and tendency direction on the horizontal axis. Infusion volume, transfusions, and MRI epidural abscess are shown to provide significant contributions to the model's output, but X-ray bone bridge and X-ray and MRI-paravertebral abscess have a moderate to low influence.

We generated a SHAP summary plot for the clinical outcome to illustrate the importance and direction of each predictive variable. The variables are sorted by their relative importance on the *y*-axis. Using the best prediction model, we calculated the SHAP value for each feature and represented them in violin charts, indicating their direction and significance to the overall outcome risk. The color on the plot represents the significance of each variable, with red indicating a higher predictor value and blue representing a lower value. For categorical variables, red denotes the presence of a specific category of the predictor, while blue indicates its absence. On the *x*-axis, each data point's position reflects the contribution of individual participants to the overall SHAP value, with high positive contributions on the far right. These findings provide insights into both patient-level and cohort-level risk factors (Fig. 8B, C). As depicted in Fig. 8B, the top five risk factors contributing to prolonged LOS are infusion volume, transfusions, MRI epidural abscess, C-reactive protein (CRP), and CT vertebral destruction.

**Fig. 9:**
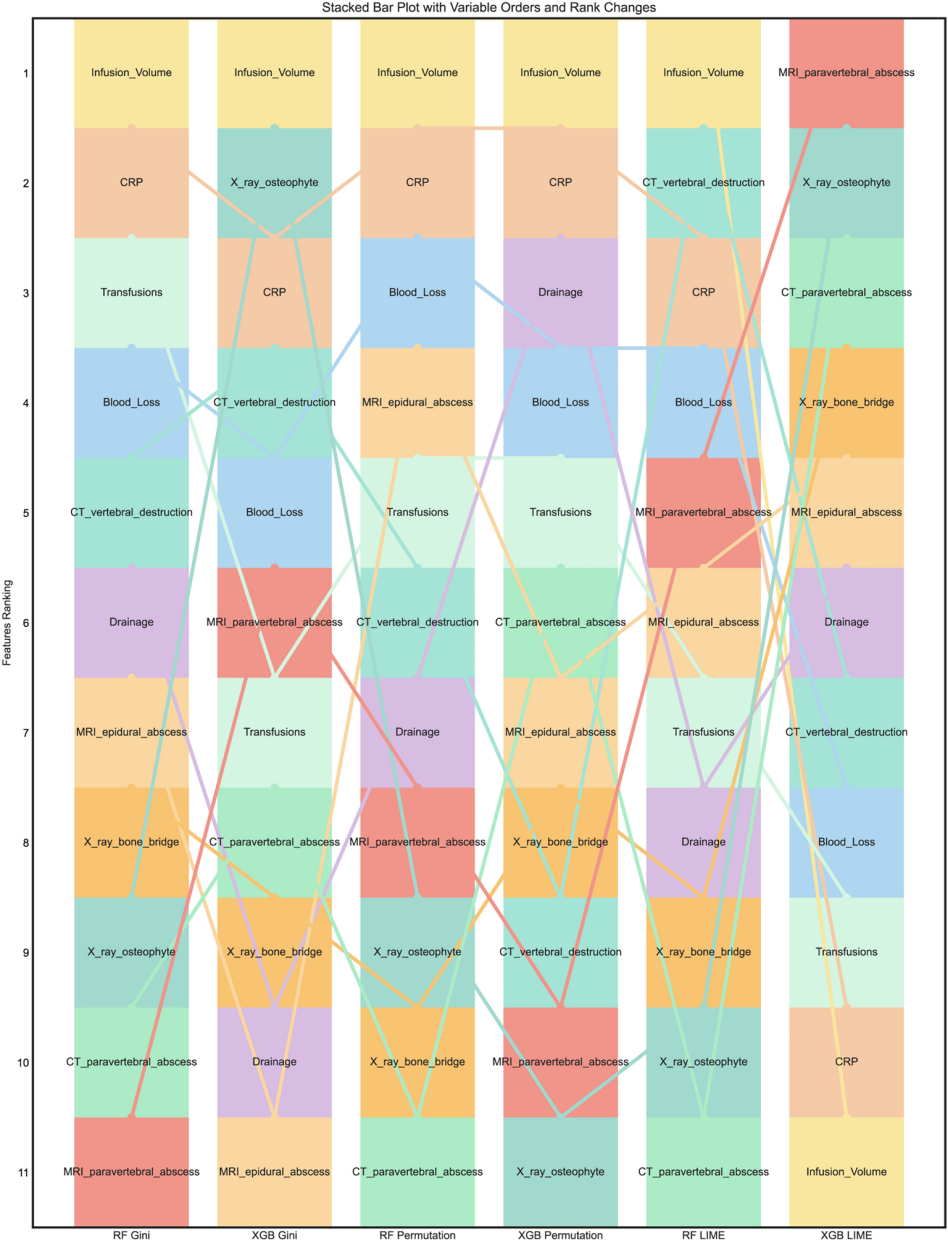
List of variables ranked by their importance to prolonged LOS with different combinations of model and importance calculation algorithm

Stacked bar plots showing the relative significance and distribution of the selected variables, using *Gini*, permutation importance (PFI), and LIME algorithm, with ranks ordered from bottom to top by their decreasing proportion of relative importance (Fig. 9). Similarly, infusion volume, CRP, CT-vertebral destruction, and transfusions are still the top features among most combinations of algorithms and important calculation strategies.

**Fig. 10:**
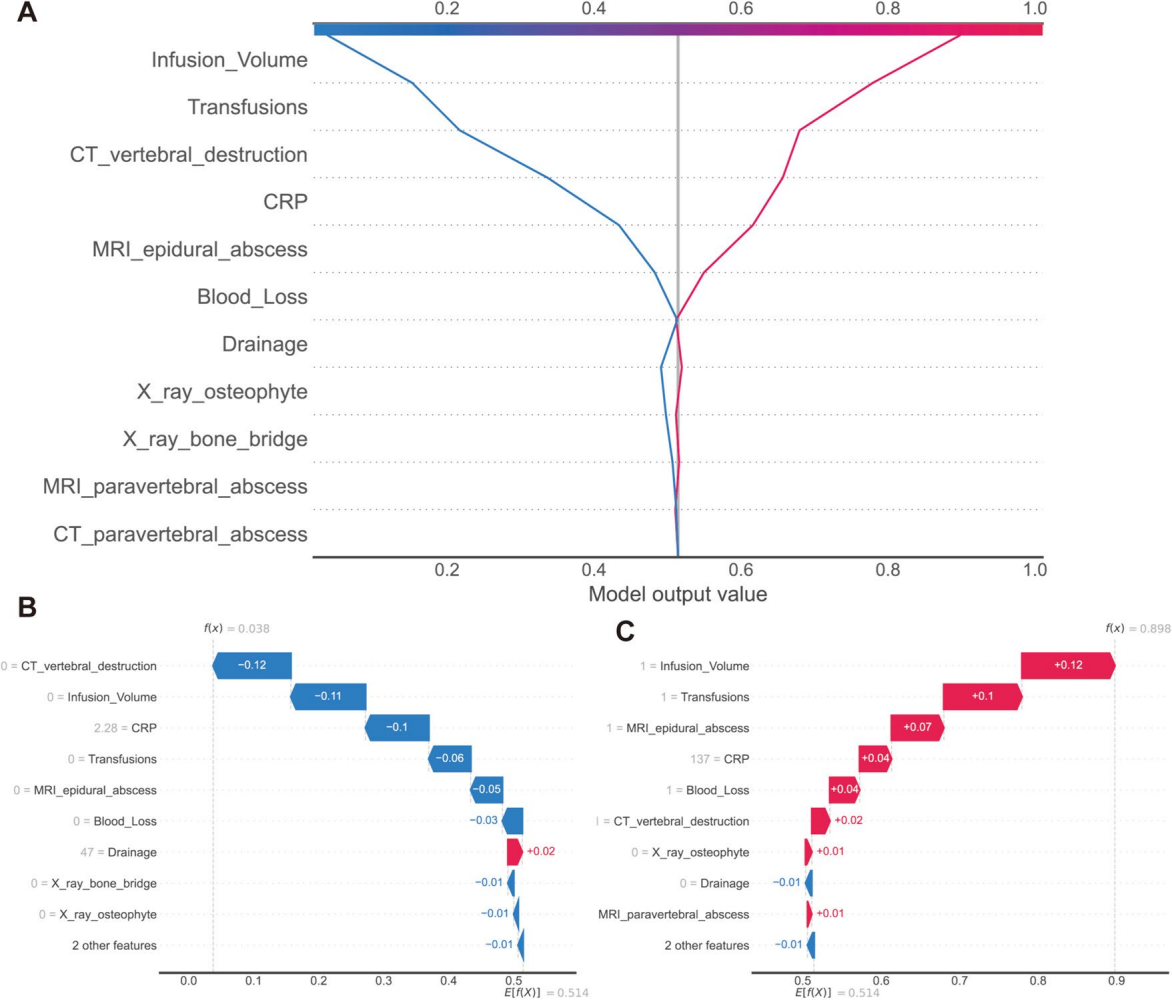
Personalized interpretation. Two patients from the testing set were shown in (**A**) SHAP decision plots; one was predicted to have “normal LOS” and the other to have “prolonged LOS”. Important features in the XGBoost model correlate to features with a high SHAP value, whereas features with a low SHAP value have a detrimental effect on the model’s output. On the other hand, characteristics with a positive value support the desired result (prolonged LOS). **B**, **C** SHAP waterfall diagrams for the two TS patients from our dataset who were selected at random. The red depicts the feature’s beneficial effects, whereas the blue hue depicts its detrimental effects. Positive qualities led to the category of extended LOS, whilst negative elements contributed to the category of normal LOS

We also used the SHAP decision plot and Waterfall plot to display the effect of each of the variables for individual predictions (Fig. 10). The decision plot shows the how XGBoost model arrives at its predictions regarding two patients (Fig. 10A). The assessment of a single patient might be interpreted using the waterfall plot. Every prediction began with the base value (0.514), which was the average SHAP value of all forecasts, and it represented the SHAP value of a characteristic as a force to raise or reduce the assessment. How much (in percentage) a certain characteristic contributed to the SHAP value depended on the length of the arrow. The color of the arrow indicated whether the contributions were constructive (red) or destructive (blue).

Elderly patients that experienced larger infusion volume, blood loss, transfusion, epidural abscess, and higher CRP underwent extended LOS as the patient with the higher SHAP value (i.e., 0.898) (Fig. 10B). However, the patient with the lower SHAP score (i.e., 0.038) did not have increased blood loss, had a normal amount of infusion volume, or underwent a transfusion (Fig. 10C).

### Online bedside tool deployment

**Fig. 11:**
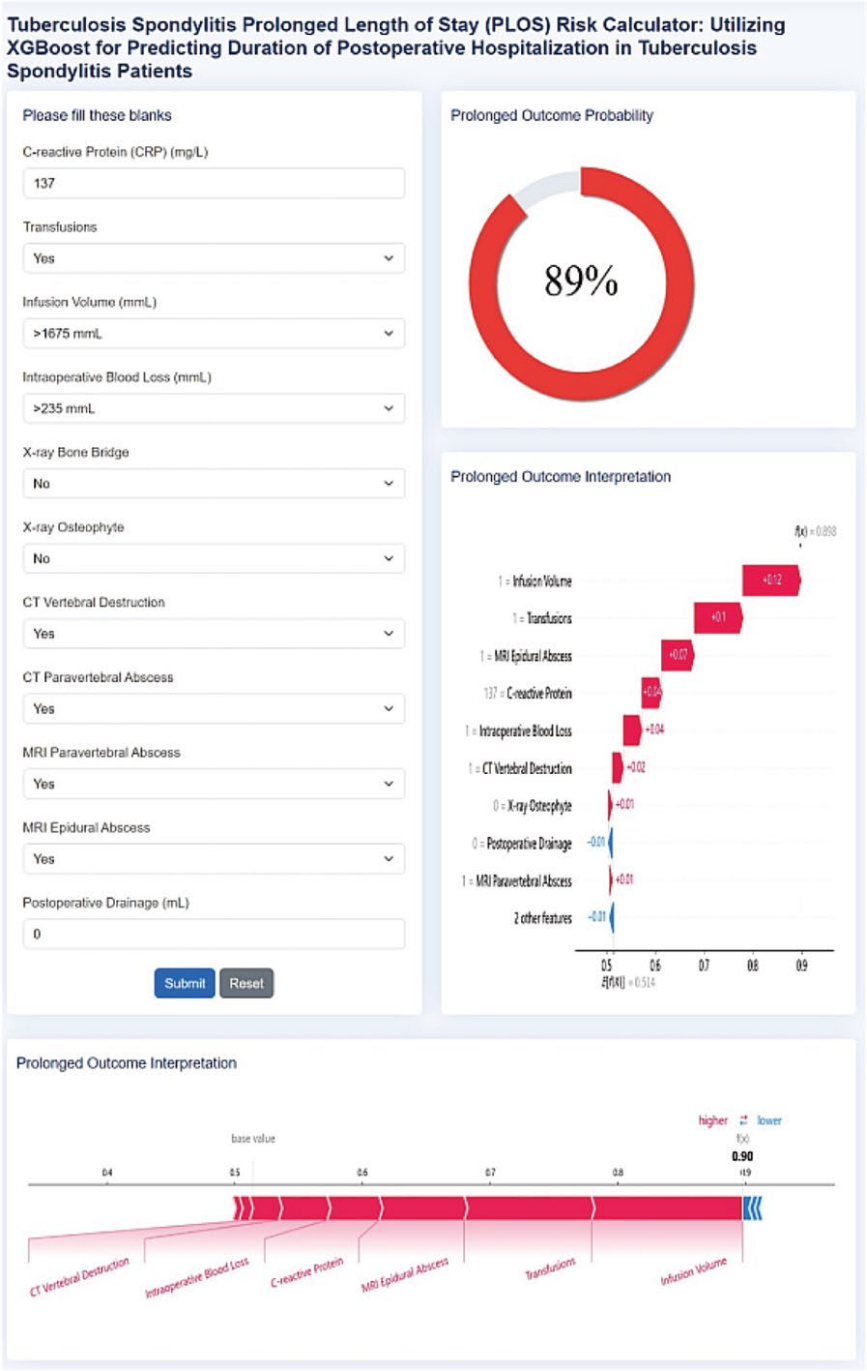
Screenshots of online free web application user interface

The final XGBoost model we chose was integrated into a web application (Fig. 11) that assesses a person's risk based on input predictors. It may determine the likelihood that TS patients would experience a longer LOS and display that information on calibration plots to define explicit visualizations. Online access to the web application is available at http://43.143.217.126:9090/tsplos.

## Discussion

Our study focused on tuberculosis spondylitis (TS), which is a form of skeletal tuberculosis that primarily affects the spine. TS, also known as Pott’s disease, is caused by the bacteria *Mycobacterium* tuberculosis and is characterized by the infection and destruction of the vertebral bodies and adjacent structures. The infection leads to inflammation and damage to the vertebral bones, intervertebral discs, and surrounding tissues, resulting in severe pain, deformity, and neurological complications. Spine surgery is often employed as a treatment option for patients with TS, aiming to alleviate pain, stabilize the spine, and prevent further neurological deterioration. However, this surgical intervention can be associated with prolonged hospital stays, which increase healthcare costs and pose challenges for patient management and resource allocation. Identifying factors that contribute to extended hospital stays after spine surgery in patients with TS is crucial for optimizing treatment strategies and improving patient outcomes. Therefore, in our study, we aimed to develop a machine learning model that could predict the likelihood of extended hospitalization, providing insights into risk factors and aiding clinicians in planning postoperative care and resource allocation effectively. Our study successfully developed an interpretable machine learning model using XGBoost to predict extended hospital stays after spine surgery in patients with tuberculosis spondylitis (TS), commonly known as Pott’s disease. We identified 11 key features, including CRP, transfusions, infusion volume, blood loss, X-ray bone bridge, X-ray osteophyte, CT-vertebral destruction, CT-paravertebral abscess, MRI-paravertebral abscess, MRI-epidural abscess, and postoperative drainage. The XGBoost model demonstrated superior performance with an AUC value of 0.86 and a low Brier Score of 0.126. By employing SHAP and LIME techniques, we were able to interpret the variables’ contributions to the predicted outcomes. Our findings not only provide accurate predictions of extended hospital stays but also contribute to identifying risk factors for future treatments. The web application deployed with the XGBoost model holds potential for clinical research applications.

Since the 1940s, when neural networks were first introduced, there has been an enormous surge in research on artificial intelligence (AI) and machine learning (ML). Computational resources, particularly in the last ten years, have gotten more affordable and available. Since more tools and libraries are now readily available, cutting-edge solutions like deep learning (DL) have emerged as a result of this development, democratizing machine learning techniques across a range of industries. For instance, DL techniques outperform conventional algorithms for the image or natural language processing, and they are frequently usable even by subject-matter experts without prior ML training [43, 44]. The conventional structure of a neural network consists of several layers connected by many nonlinear tangled interactions. It is impossible to fully understand how the neural network arrived at its choice, even if one were to examine all of these levels and characterize their relationships. There is growing worry that these black boxes may be biased in some way and that this bias may go unnoticed in several application domains. This can have significant ramifications, particularly in medical applications [45, 46]. Making medical decisions involves a lot of risk. It should come as no surprise that medical professionals are concerned about the black box aspect of deep learning. To address this problem, explainable artificial intelligence (XAI) has been proposed to allow people to understand and explain the decision-making process of machine learning models. XAI technology is a new and emerging field in AI that aims to explain how AI algorithms work and generate complex decisions by fundamentally investigating the causes and decision rules of AI algorithms [47–49].

In our research, we used a model explanation algorithm, and the result shows the identification and understanding of hazardous influences linked to prolonged hospital stays following surgery for tuberculosis spondylitis are crucial for enhancing patient outcomes and optimizing healthcare resources. CRP, a commonly used marker for inflammation, elevated CRP levels post-surgery often implies presence of infection or inflammation. In tuberculosis spondylitis patients, this could lead to delayed recovery and prolonged hospitalization [50]. Although transfusions reduce blood loss after surgery, there are hazards associated with them, including infections and transfusion reactions. These issues may make it more difficult for the patient to heal, requiring extended hospital stays for care and observation [51]. Proper fluid management is vital for maintaining organ function. Both excessive and insufficient infusion volumes can potentially prolong hospitalization [52]. Significant intraoperative blood loss can result in post-operative anemia and compromised tissue perfusion, delaying the patient’s recovery. In addition, blood loss increases the risk of post-operative infections, further extending the hospitalization period. Exitance of X-ray bone bridge and osteophytes implies chronicity of the disease and may indicate a more advanced stage of tuberculosis spondylitis. These could make the surgical procedure more complex, subsequently result in prolonged hospitalization [53]. CT-vertebral destruction indicates the extent of vertebral damage. Severe destruction of the vertebral bodies requires more extensive surgical interventions. This can prolong the surgery time and subsequently impact the length of stay in the hospital [54]. CT-paravertebral abscess and MRI-paravertebral abscess refer to the presence of abscesses or collections of pus in the tissues surrounding the affected vertebrae. Treatment typically requires drainage of the abscess, which may require additional procedures and postoperative care, may lead to extended hospitalization. MRI-epidural abscess, which indicates the presence of abscess in the epidural space, where is the area around the spinal cord. Epidural abscesses can compress the spinal cord or nerve roots, causes neurological deficits. The involvement of the epidural space can significantly impact the length of stay in the hospital due to the need for close monitoring and management of neurological symptoms. Implementation of postoperative drainage may increase complications such as bleeding, infection at the surgical site, and catheter-related bloodstream infections, these may responsible for prolonged length of hospital stay for tuberculosis spondylitis patients.

The distribution of class unbalance is a typical issue for real-world medical data [55]. The number of observations for each class or label is not equal-it is called unbalanced data, which is particularly common in the machine learning field [56]. The unequal quality of the majority and minority classes is one of the key issues with employing data analysis for diagnosis and therapy, and it can present challenges for predictive modeling [57]. The most common issue brought on by data unbalanced categorization is incorrect diagnoses of the minority class, which is more likely and cost-sensitive than the majority class [58]. The accuracy of the general categorization is optimized by many machine learning methods. This design idea, however, leads to mistakes in the minority class’s categorization [59]. The method, in general, makes a bigger contribution to the higher quality classification of the majority class samples, which are thought to be more important. Standard machine learning with unbalanced data might result in a decision boundary that is heavily skewed by the majority class, resulting in low precision for the minority class. Ultimately, improving the classification performance for the minority class is the aim of tackling the unbalanced data problem. Unbalanced data can have significant effects on model output, one of which is that it may cause the model to give the dominant class priority over the minority class. The model thus exhibits a bias in favor of the majority class, resulting in poor results for the minority class and deceptively high overall accuracy. In addition, common assessment measures like accuracy could not correctly represent how well a model performs with an imbalanced dataset [60, 61]. Several strategies have been suggested to reduce the effect of unbalanced data on model output. The two primary types of these methods are algorithmic-level methods and data-level methods. Algorithmic level techniques change the learning algorithm itself to manage imbalanced data [62, 63]. On the other hand, the data-level balancing strategy has been frequently utilized to balance training samples across the available techniques [64]. It has also been frequently used to balance data using a loss-based (cost-sensitive) method that gives minority samples more weight than majority ones. The classifier-design strategy for balancing involves creating computational strategies that are integrated into a classifier to automatically address the class-imbalance problem [62].

To provide each class with a fairer representation, data-level procedures rebalance or expand the dataset. Undersampling the majority class by randomly omitting certain data is one strategy. This method shrinks the dataset but could potentially abandon valuable data. Another strategy is to generate fake samples or duplicate observations to oversample the minority class. SMOTE is the most used oversampling approach [16, 63]. SMOTE (Synthetic Minority Over-sampling Technique), is a popular data augmentation technique in oversampling. By interpolating between the feature space of two or more nearest nearby samples, SMOTE creates synthetic fake samples of the minority class. The difference between two feature vectors in the minority class is added to one or the other to create a new synthetic point if a point in the minority class is isolated. In this procedure, a minority class observation is chosen at random, and its k nearest neighbors are identified using feature space distance metrics such as the Euclidean distance or Manhattan distance [15, 65]. Compared to other sampling techniques, SMOTE has a number of advantages. Creating synthetic samples reduces the chance of overfitting the training data and is computationally efficient, because it can quickly process big datasets and create many synthetic examples. SMOTE does not introduce any noise to the dataset and also maintains the minority class’s original distribution. SMOTE can also decrease the likelihood of the model overfitting to a few particular minority class data while increasing the generalizability and robustness of the model [66, 67].

Artificial intelligence (AI) has accelerated the evolution of medical practice. Recent developments in machine learning, digital data collection, and computer infrastructure have made it possible for AI applications to proliferate in sectors that were previously thought to be the exclusive purview of human knowledge [68]. The application of AI at the bedside has several advantages, including reliving the workload of healthcare workers, splitting up the workload of healthcare practitioners, replacing repetitive and requiring little cognitive tasks, and augmenting clinical practice. However, the practical ability of the AI model at the bedside has several challenges to be addressed [69]. The first challenge is data availability, Electronic health records (EHRs) are frequently kept in healthcare facilities and contain an abundance of data about a patient's medical history. The information might not be standardized, fragmented, or in a format that AI models can simply process. In addition, using personal data raises a number of privacy and ethical issues, and different jurisdictions might have varying standards for data access [70, 71]. The lack of interpretability and transparency presents another difficulty when using AI models in clinical settings. Clinicians may find it challenging to comprehend how AI models generate their recommendations due to their complexity. Because of this model’s lack of interpretability, doctors may be wary of it and be less likely to trust these tools in actual clinical settings [72, 73]. Clinical practice might be revolutionized by AI models, which could also lead to better patient outcomes. Their practical applicability and accompanying difficulties must be carefully considered in order for them to be successfully incorporated into routine clinical practice. To overcome these obstacles, it is necessary for physicians, researchers, and developers to work together to build AI models that are compatible with clinical procedures and enhance patient safety [68].

With the advent of web applications for AI models, there is now a great opportunity to expand the reach and impact of AI in medicine. Web applications of AI models have several benefits for healthcare workers, these are: faster and more accurate diagnosis, improved efficiency and workflow, better disease prevention and management, personalized healthcare, and enhanced medical research. These can significantly improve the accuracy and speed of diagnoses, provide personalized medical care, and enhance research efforts. In addition, they can help reduce costs, optimize workflow, and improve patient outcomes [74–76]. In this study, we have built a web application with the aim of bedside utilization, which can save some effort and bring benefits to clinical practice.

The developed prediction model offers valuable insights that can be integrated into existing clinical workflows and decision-making processes. By leveraging the model's predictions to personalize care planning, allocate resources effectively, stratify patient risks, enhance discharge planning, and support clinical decision-making, healthcare providers can optimize patient outcomes, improve resource utilization, and enhance the overall quality of care delivery for tuberculosis spondylitis patients undergoing surgery.

## Limitations

It is important to note the limitations of this study. First off, the study’s sample size was quite small, which would restrict how broadly the results can be applied. A larger and more diverse sample would enhance the reliability and external validity of the results. Second, the quality and availability of the data collected could have influenced the accuracy and robustness of the predictive model. Retrospective or incomplete data may introduce biases or affect the model’s performance. Third, external validation of the model was not conducted using an independent dataset. Without external validation, it is challenging to ascertain the model's applicability to different populations or healthcare settings. Fourth, the selection of variables in the model may be incomplete. It is possible that other relevant variables were overlooked, and including them could improve the predictive power of the model. Fifth, due to retrospective study from one -single institution, selection bias, institutional bias, and patient diversity exist in our study, further large sample, multicenter studies need to tackle these problems. Sixth, missing data could lead to bias, reduced sample size and imputation uncertainty, in future research, we aim to further explore the impact of missing data. Lastly, while the study employed SHAP and LIME techniques to interpret the variables’ contributions to the predicted outcomes, machine learning models are inherently black-box, making it challenging to fully understand the underlying mechanisms and causal relationships. To fully comprehend the causes causing prolonged hospital admissions in patients with TB spondylitis, more studies may be required. It is critical to recognize these limitations, because they open up possibilities for future study and might lead to advancements in the creation of prediction models for prolonged hospital stays in TB spondylitis patients. These limitations highlight opportunities for future research to address these issues and enhance the development of predictive models in this context.

## Conclusion

In this study, we utilized explainable artificial intelligence (XAI) techniques, such as SHAP and LIME, to predict prolonged hospital stays after surgery for tuberculosis spondylitis patients. Furthermore, the unbalanced data was the deal was manipulated via SMOTE technique. The model incorporated preoperative clinical features and examination results to forecast the duration of hospitalization, resulting in high accuracy and predictive capability. This model offers crucial decision support for healthcare professionals and insights to improve surgical outcomes and reduce hospital stay duration for these patients with the online web application.

### Supplementary Information

Supplementary Material 1.

## Data Availability

Upon a reasonable request, the corresponding authors of this article will provide unrestricted access to the original data.

## References

[1] Lener S, Hartmann S, Barbagallo GMV, Certo F, Thome C, Tschugg A. Management of spinal infection: a review of the literature. Acta Neurochir (Wien). 2018;160(3):487–96. 29356895 10.1007/s00701-018-3467-2 PMC5807463

[2] Trecarichi EM, Di Meco E, Mazzotta V, Fantoni M. Tuberculous spondylodiscitis: epidemiology, clinical features, treatment, and outcome. Eur Rev Med Pharmacol Sci. 2012;16(Suppl 2):58–72. 22655484

[3] Arockiaraj J, Balaji GS, Cherian VM, et al. Drug resistant skeletal tuberculosis in a tertiary care centre in South India. J Clin Orthop Trauma. 2018;9:S44–8. 10.1016/j.jcot.2017.12.009 PMC5883913 29628698

[4] Assaghir YM, Refae HH, Alam-Eddin M. Anterior versus posterior debridement fusion for single-level dorsal tuberculosis: the role of graft-type and level of fixation on determining the outcome. Eur Spine J. 2016;25(12):3884–93. 26988554 10.1007/s00586-016-4516-2

[5] Moon MS. Tuberculosis of spine: current views in diagnosis and management. Asian Spine J. 2014;8(1):97–111. 24596613 10.4184/asj.2014.8.1.97 PMC3939378

[6] García-Romero A, Escribano Á, Tribó JA. The impact of health research on length of stay in Spanish public hospitals. Res Policy. 2017;46(3):591–604. 10.1016/j.respol.2017.01.006

[7] Waseem M, Prasankumar R, Pagan K, Leber M. A retrospective look at length of stay for pediatric psychiatric patients in an urban emergency department. Pediatr Emerg Care. 2011;27(3):170–3. 21346682 10.1097/PEC.0b013e31820d644b

[8] Gruskay JA, Fu M, Bohl DD, Webb ML, Grauer JN. Factors affecting length of stay after elective posterior lumbar spine surgery: a multivariate analysis. Spine J. 2015;15(6):1188–95. 24184639 10.1016/j.spinee.2013.10.022

[9] Debono B, Corniola MV, Pietton R, Sabatier P, Hamel O, Tessitore E. Benefits of enhanced recovery after surgery for fusion in degenerative spine surgery: impact on outcome, length of stay, and patient satisfaction. Neurosurg Focus. 2019;46(4):E6. 10.3171/2019.1.FOCUS18669 30933923

[10] Carbonell JG, Michalski RS, Mitchell TM. 1—an overview of machine learning. In: Michalski RS, Carbonell JG, Mitchell TM, editors. Machine Learning. San Francisco: Morgan Kaufmann; 1983. p. 3–23.

[11] Rai A. Explainable AI: from black box to glass box. J Acad Mark Sci. 2020;48(1):137–41. 10.1007/s11747-019-00710-5

[12] Neves I, Folgado D, Santos S, et al. Interpretable heartbeat classification using local model-agnostic explanations on ECGs. Comput Biol Med. 2021;133: 104393. 33915362 10.1016/j.compbiomed.2021.104393

[13] Slack D, Hilgard S, Jia E, Singh S, Lakkaraju H. Fooling LIME and SHAP: adversarial attacks on post hoc explanation methods. Proceedings of the AAAI/ACM conference on AI, ethics, and society; 2020; New York, NY, USA.

[14] Gao M, Sun J, Jiang Z, et al. Comparison of tuberculous and brucellar spondylitis on magnetic resonance images. Spine (Phila Pa 1976). 2017;42(2):113–21. 27196025 10.1097/BRS.0000000000001697

[15] Xu Z, Shen D, Nie T, Kou Y. A hybrid sampling algorithm combining M-SMOTE and ENN based on Random forest for medical imbalanced data. J Biomed Inform. 2020;107: 103465. 32512209 10.1016/j.jbi.2020.103465

[16] Blagus R, Lusa L. SMOTE for high-dimensional class-imbalanced data. BMC Bioinform. 2013;14:106. 10.1186/1471-2105-14-106 PMC3648438 23522326

[17] Na KS. Prediction of future cognitive impairment among the community elderly: a machine-learning based approach. Sci Rep. 2019;9(1):3335. 30833698 10.1038/s41598-019-39478-7 PMC6399248

[18] de Belen RAJ, Bednarz T, Sowmya A, Del Favero D. Computer vision in autism spectrum disorder research: a systematic review of published studies from 2009 to 2019. Transl Psychiatry. 2020;10(1):333. 32999273 10.1038/s41398-020-01015-w PMC7528087

[19] Shim M, Lee SH, Hwang HJ. Inflated prediction accuracy of neuropsychiatric biomarkers caused by data leakage in feature selection. Sci Rep. 2021;11(1):7980. 33846489 10.1038/s41598-021-87157-3 PMC8042090

[20] Azur MJ, Stuart EA, Frangakis C, Leaf PJ. Multiple imputation by chained equations: what is it and how does it work? Int J Methods Psychiatr Res. 2011;20(1):40–9. 21499542 10.1002/mpr.329 PMC3074241

[21] Tibshirani R. The lasso method for variable selection in the Cox model. Stat Med. 1997;16(4):385–95. 9044528 10.1002/(SICI)1097-0258(19970228)16:4<385::AID-SIM380>3.0.CO;2-3

[22] Huang X, Zhang L, Wang B, Li F, Zhang Z. Feature clustering based support vector machine recursive feature elimination for gene selection. Appl Intell. 2018;48(3):594–607. 10.1007/s10489-017-0992-2

[23] Sanz H, Valim C, Vegas E, Oller JM, Reverter F. SVM-RFE: selection and visualization of the most relevant features through non-linear kernels. BMC Bioinform. 2018;19(1):432. 10.1186/s12859-018-2451-4 PMC6245920 30453885

[24] Nembrini S, Konig IR, Wright MN. The revival of the Gini importance? Bioinformatics. 2018;34(21):3711–8. 29757357 10.1093/bioinformatics/bty373 PMC6198850

[25] Altmann A, Tolosi L, Sander O, Lengauer T. Permutation importance: a corrected feature importance measure. Bioinformatics. 2010;26(10):1340–7. 20385727 10.1093/bioinformatics/btq134

[26] Marin D, Tang M, Ayed IB, Boykov Y. Kernel clustering: density biases and solutions. IEEE Trans Pattern Anal Mach Intell. 2019;41(1):136–47. 29990278 10.1109/TPAMI.2017.2780166

[27] Breiman L. Random forests. Mach Learn. 2001;45(1):5–32. 10.1023/A:1010933404324

[28] Tsuzuki S, Fujitsuka N, Horiuchi K, et al. Factors associated with sufficient knowledge of antibiotics and antimicrobial resistance in the Japanese general population. Sci Rep. 2020;10(1):3502. 32103110 10.1038/s41598-020-60444-1 PMC7044168

[29] Chen S, Webb GI, Liu L, Ma X. A novel selective naïve Bayes algorithm. Knowl-Based Syst. 2020;192: 105361. 10.1016/j.knosys.2019.105361

[30] Sufriyana H, Husnayain A, Chen Y-L, et al. Comparison of multivariable logistic regression and other machine learning algorithms for prognostic prediction studies in pregnancy care: systematic review and meta-analysis. JMIR Med Inform. 2020;8(11): e16503. 33200995 10.2196/16503 PMC7708089

[31] Yasin P, Mardan M, Xu T, et al. Development and validation of a diagnostic model for differentiating tuberculous spondylitis from brucellar spondylitis using machine learning: a retrospective cohort study. Front Surg. 2022;9: 955761. 36684365 10.3389/fsurg.2022.955761 PMC9852539

[32] Sarkar M, Leong TY. Application of K-nearest neighbors algorithm on breast cancer diagnosis problem. *Proc AMIA Symp.* 2000. p. 759–63. PMC2243774 11079986

[33] Probst P, Wright MN, Boulesteix AL. Hyperparameters and tuning strategies for random forest. Wires Data Min Knowl. 2019;9(3): e1301. 10.1002/widm.1301

[34] Noble WS. What is a support vector machine? Nat Biotechnol. 2006;24(12):1565–7. 17160063 10.1038/nbt1206-1565

[35] Song YY, Lu Y. Decision tree methods: applications for classification and prediction. Shanghai Arch Psychiatry. 2015;27(2):130–5. 26120265 10.11919/j.issn.1002-0829.215044 PMC4466856

[36] Torlay L, Perrone-Bertolotti M, Thomas E, Baciu M. Machine learning–XGBoost analysis of language networks to classify patients with epilepsy. Brain Inform. 2017;4(3):159–69. 28434153 10.1007/s40708-017-0065-7 PMC5563301

[37] Shehadeh A, Alshboul O, Al Mamlook RE, Hamedat O. Machine learning models for predicting the residual value of heavy construction equipment: an evaluation of modified decision tree, LightGBM, and XGBoost regression. Autom Constr. 2021;129: 103827. 10.1016/j.autcon.2021.103827

[38] Parvandeh S, Yeh H-W, Paulus MP, McKinney BA. Consensus features nested cross-validation. Bioinformatics. 2020;36(10):3093–8. 31985777 10.1093/bioinformatics/btaa046 PMC7776094

[39] Mangalathu S, Hwang S-H, Jeon J-S. Failure mode and effects analysis of RC members based on machine-learning-based SHapley Additive exPlanations (SHAP) approach. Eng Struct. 2020;219: 110927. 10.1016/j.engstruct.2020.110927

[40] Wen X, Xie Y, Wu L, Jiang L. Quantifying and comparing the effects of key risk factors on various types of roadway segment crashes with LightGBM and SHAP. Accid Anal Prev. 2021;159: 106261. 34182322 10.1016/j.aap.2021.106261

[41] Ribeiro MT, Singh S, Guestrin C. “Why Should I Trust You?”: explaining the predictions of any classifier. In: Proceedings of the 22nd ACM SIGKDD international conference on knowledge discovery and data mining; 2016; San Francisco, California, USA.

[42] Silva LB, Jimenez RC, Blomberg N, Luis OJ. General guidelines for biomedical software development. F1000Res. 2017;6:273. 28443186 10.12688/f1000research.10750.2 PMC5383938

[43] Spinner T, Schlegel U, Schafer H, El-Assady M. explAIner: a visual analytics framework for interactive and explainable machine learning. IEEE Trans Vis Comput Graph. 2020;26(1):1064–74. 31442998 10.1109/TVCG.2019.2934629

[44] Hohman FM, Kahng M, Pienta R, Chau DH. Visual analytics in deep learning: an interrogative survey for the next frontiers. IEEE Trans Vis Comput Graph. 2018;25:2674–93. 10.1109/TVCG.2018.2843369 PMC6703958 29993551

[45] van der Velden BHM, Kuijf HJ, Gilhuijs KGA, Viergever MA. Explainable artificial intelligence (XAI) in deep learning-based medical image analysis. Med Image Anal. 2022;79: 102470. 35576821 10.1016/j.media.2022.102470

[46] Murdoch WJ, Singh C, Kumbier K, Abbasi-Asl R, Yu B. Definitions, methods, and applications in interpretable machine learning. Proc Natl Acad Sci U S A. 2019;116(44):22071–80. 31619572 10.1073/pnas.1900654116 PMC6825274

[47] Jia X, Ren L, Cai J. Clinical implementation of AI technologies will require interpretable AI models. Med Phys. 2020;47(1):1–4. 31663612 10.1002/mp.13891

[48] Litjens G, Kooi T, Bejnordi BE, et al. A survey on deep learning in medical image analysis. Med Image Anal. 2017;42:60–88. 28778026 10.1016/j.media.2017.07.005

[49] Meijering E. A bird’s-eye view of deep learning in bioimage analysis. Comput Struct Biotechnol J. 2020;18:2312–25. 32994890 10.1016/j.csbj.2020.08.003 PMC7494605

[50] Sudprasert W, Piyapromdee U, Lewsirirat S. Neurological recovery determined by C-reactive protein, erythrocyte sedimentation rate and two different posterior decompressive surgical procedures: a retrospective clinical study of patients with spinal tuberculosis. J Med Assoc Thailand Chotmaihet thangphaet. 2015;98(10):993–1000. 26638591

[51] Chen L, Gan Z, Huang S, et al. Blood transfusion risk prediction in spinal tuberculosis surgery: development and assessment of a novel predictive nomogram. BMC Musculoskelet Disord. 2022;23(1):182. 35216570 10.1186/s12891-022-05132-z PMC8876452

[52] Child DL, Cao Z, Seiberlich LE, et al. The costs of fluid overload in the adult intensive care unit: is a small-volume infusion model a proactive solution? ClinicoEcon Outcomes Res CEOR. 2015;7:1–8. 10.2147/CEOR.S72776 PMC4271789 25548524

[53] Esteves S, Catarino I, Lopes D, Sousa CJJS. Spinal tuberculosis: rethinking an old disease. J Spine. 2017;6(1):358–66. 10.4172/2165-7939.1000358

[54] De Backer A, Mortelé K, Vanschoubroeck I, et al. Tuberculosis of the spine: CT and MR imaging features. JBR-BTR. 2005;88(2):92–7. 15906583

[55] Diz J, Marreiros G, Freitas A. Applying data mining techniques to improve breast cancer diagnosis. J Med Syst. 2016;40(9):203. 27498205 10.1007/s10916-016-0561-y

[56] Fotouhi S, Asadi S, Kattan MW. A comprehensive data level analysis for cancer diagnosis on imbalanced data. J Biomed Inform. 2019;90: 103089. 30611011 10.1016/j.jbi.2018.12.003

[57] Majid A, Ali S, Iqbal M, Kausar N. Prediction of human breast and colon cancers from imbalanced data using nearest neighbor and support vector machines. Comput Methods Programs Biomed. 2014;113(3):792–808. 24472367 10.1016/j.cmpb.2014.01.001

[58] Lin WJ, Chen JJ. Class-imbalanced classifiers for high-dimensional data. Brief Bioinform. 2013;14(1):13–26. 22408190 10.1093/bib/bbs006

[59] Li Y, Hsu WW. A classification for complex imbalanced data in disease screening and early diagnosis. Stat Med. 2022;41(19):3679–95. 35603639 10.1002/sim.9442 PMC9541048

[60] Bria A, Karssemeijer N, Tortorella F. Learning from unbalanced data: a cascade-based approach for detecting clustered microcalcifications. Med Image Anal. 2014;18(2):241–52. 24292553 10.1016/j.media.2013.10.014

[61] Duan F, Zhang S, Yan Y, Cai Z. An oversampling method of unbalanced data for mechanical fault diagnosis based on mean radius-SMOTE. Sensors (Basel). 2022;22(14):5166. 35890845 10.3390/s22145166 PMC9324964

[62] Choi HS, Jung D, Kim S, Yoon S. Imbalanced data classification via cooperative interaction between classifier and generator. IEEE Trans Neural Netw Learn Syst. 2022;33(8):3343–56. 33531305 10.1109/TNNLS.2021.3052243

[63] Ma L, Fan S. CURE-SMOTE algorithm and hybrid algorithm for feature selection and parameter optimization based on random forests. BMC Bioinformatics. 2017;18(1):169. 28292263 10.1186/s12859-017-1578-z PMC5351181

[64] Nakamura M, Kajiwara Y, Otsuka A, Kimura H. LVQ-SMOTE—learning vector quantization based synthetic minority over-sampling technique for biomedical data. BioData Min. 2013;6(1):16. 24088532 10.1186/1756-0381-6-16 PMC4016036

[65] Dablain D, Krawczyk B, Chawla NV. DeepSMOTE: fusing deep learning and SMOTE for imbalanced data. IEEE Trans Neural Netw Learn Syst. 2022;34:6390–404. 10.1109/TNNLS.2021.3136503 35085094

[66] Sreejith S, Khanna Nehemiah H, Kannan A. Clinical data classification using an enhanced SMOTE and chaotic evolutionary feature selection. Comput Biol Med. 2020;126: 103991. 32987205 10.1016/j.compbiomed.2020.103991

[67] Xu Z, Shen D, Kou Y, Nie T. A synthetic minority oversampling technique based on gaussian mixture model filtering for imbalanced data classification. IEEE Trans Neural Netw Learn Syst. 2022;35:3740–53. 10.1109/TNNLS.2022.3197156 35984792

[68] Yu KH, Beam AL, Kohane IS. Artificial intelligence in healthcare. Nat Biomed Eng. 2018;2(10):719–31. 31015651 10.1038/s41551-018-0305-z

[69] Aung YYM, Wong DCS, Ting DSW. The promise of artificial intelligence: a review of the opportunities and challenges of artificial intelligence in healthcare. Br Med Bull. 2021;139(1):4–15. 34405854 10.1093/bmb/ldab016

[70] Dilsizian SE, Siegel EL. Artificial intelligence in medicine and cardiac imaging: harnessing big data and advanced computing to provide personalized medical diagnosis and treatment. Curr Cardiol Rep. 2014;16(1):441. 24338557 10.1007/s11886-013-0441-8

[71] Johnson KW, Torres Soto J, Glicksberg BS, et al. Artificial intelligence in cardiology. J Am Coll Cardiol. 2018;71(23):2668–79. 29880128 10.1016/j.jacc.2018.03.521

[72] Wang F, Preininger A. AI in health: state of the art, challenges, and future directions. Yearb Med Inform. 2019;28(1):16–26. 31419814 10.1055/s-0039-1677908 PMC6697503

[73] Kahn CE Jr. From images to actions: opportunities for artificial intelligence in radiology. Radiology. 2017;285(3):719–20. 29155645 10.1148/radiol.2017171734

[74] Saunders CH, Sierpe A, Stevens G, et al. Co-development of a web application (COVID-19 social site) for long-term care workers (“Something for Us”): user-centered design and participatory research study. J Med Internet Res. 2022;24(9): e38359. 35926074 10.2196/38359 PMC9506501

[75] Speake C, Presnell S, Domico K, et al. An interactive web application for the dissemination of human systems immunology data. J Transl Med. 2015;13:196. 26088622 10.1186/s12967-015-0541-x PMC4474328

[76] Kavanagh ME, Chiavaroli L, Glenn AJ, et al. A web-based health application to translate nutrition therapy for cardiovascular risk reduction in primary care (PortfolioDiet.app): quality improvement and usability testing study. JMIR Hum Factors. 2022;9(2): e34704. 35451981 10.2196/34704 PMC9073604

