# Supplementary material for: Machine learning-enabled prediction of prolonged length of stay in hospital after surgery for tuberculosis spondylitis patients with unbalanced data: a novel approach using explainable artificial intelligence (XAI)
Source: Eur J Med Res. 2024 Jul 25;29:383. doi: 10.1186/s40001-024-01988-0 (PMC11270948; doi:10.1186/s40001-024-01988-0)
Supplement: Supplementary file 1 — Supplementary Material 1. [file 40001_2024_1988_MOESM1_ESM.docx]

Supplementary table 1

| **LOS** | **y_train_count** | **y_train_proportion** | **y_test_count** | **y_test_proportion** |
| --- | --- | --- | --- | --- |
|  | **Before oversampling** | | |  |
| **Normal** | 319 | 0.786 | 134 | 0.770 |
| **Prolonged** | 87 | 0.214 | 40 | 0.229 |
|  | **After oversampling** | | |  |
| **Normal** | 319 | 0.5 | 134 | 0.770 |
| **Prolonged** | 319 | 0.5 | 40 | 0.229 |

LOS, length of stay


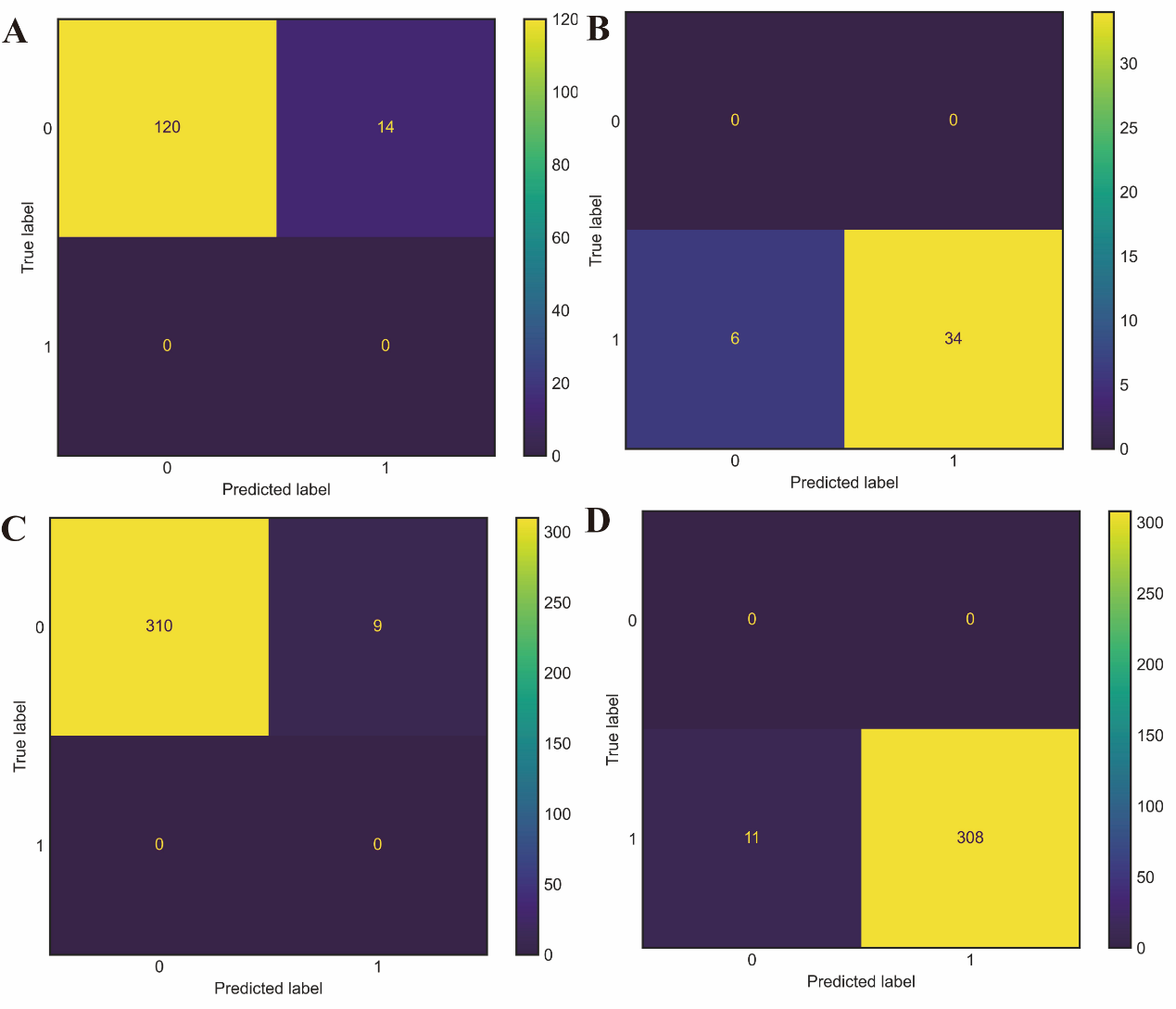


**Supplementary Figure 1** Prediction results of selected Model (XGBoost) by different groups

(A) Confusion matrix plot of the model for NLOS (normal length of stay) patients on the testing set; (B) Confusion matrix plot of the model for PLOS (prolonged length of stay) patients on the testing set; (C) Confusion matrix plot of the model for NLOS (normal length of stay) patients on the training set; (D) Confusion matrix plot of the model for PLOS (prolonged length of stay) patients on the training set.
